# Proton-Relaying Adsorbates Induce Non-Nernstian Behavior in Oxygen Reduction

**DOI:** 10.1021/acscatal.5c01767

**Published:** 2025-08-01

**Authors:** Lulu Zhang, Dongchen Zhao, Weiqiang Tang, Yanxia Chen, Jun Huang

**Affiliations:** † Hefei National Research Center for Physical Sciences at Microscale, Department of Chemical Physics, 12652 University of Science and Technology of China, Hefei 230026, China; ‡ Institute of Energy Technologies, IET-3: Theory and Computation of Energy Materials, 28334 Forschungszentrum Jülich GmbH, 52425 Jülich, Germany; § Theory of Electrocatalytic Interfaces, Faculty of Georesources and Materials Engineering, RWTH Aachen University, 52062 Aachen, Germany

**Keywords:** oxygen reduction reaction, non-Nernstian behavior, anion- and cation-dependent pH effects, hierarchical theoretical model, local reaction environment, proton-relaying role, adsorbed sulfate anions

## Abstract

Proton-coupled electron transfer (PCET) is central to energy conversion processes in fuel cells, electrolysis, and biological systems. According to the Nernst equation, the equilibrium potential of PCET shifts by around −60 mV/pH relative to the standard hydrogen electrode at room temperature. Here, we reveal significant deviations from this expected Nernstian behavior in the oxygen reduction reaction (ORR) at Pt(111) in H_2_SO_4_/M_2_SO_4_ (M = Li, Cs) solutions, with a pronounced dependence on the cation identity, whereas Nernstian behavior is retained in HClO_4_/LiClO_4_ solutions. To elucidate the origin of these pH effects, we employ a hierarchical theoretical framework that integrates density functional theory calculations, multistep microkinetic modeling, and the local reaction environment (LRE) model describingmass transport and electrical double layer effects. Our analysis uncovers a previously unrecognized mechanistic role of adsorbed sulfate anions in mediating proton transfer. Specifically, sulfate anions attract hydrated protons via electrostatic interactions, leading to the formation of adsorbed bisulfate species, which then act as proton donors in the ORR. This shift in the proton donor species explains the observed reduction in the proton reaction order from 1 in HClO_4_/LiClO_4_ to 0.5 in H_2_SO_4_/Li_2_SO_4_ and 0.75 in H_2_SO_4_/Cs_2_SO_4_ solutions. This work advances the understanding of anion- and cation-dependent pH effects in electrocatalysis by highlighting the role of LRE modulation. Furthermore, it demonstrates how a combined theoretical and computational approach can disentangle complex, multiscale interactions in electrochemical reactions.

## Graphical abstract

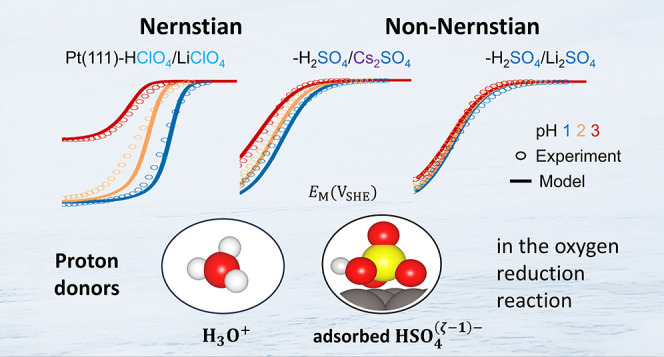

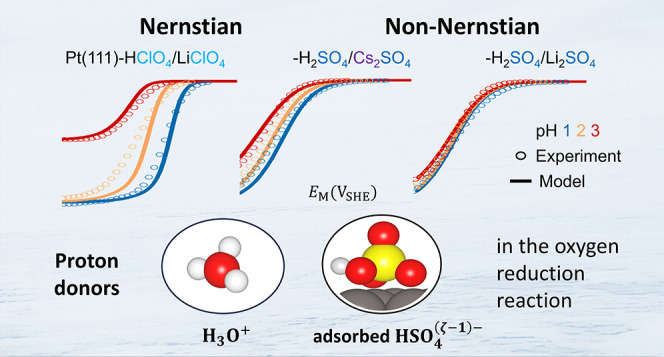

## Introduction

Many important electrocatalytic reactions belong to proton-coupled electron transfer (PCET) reactions, A+*n*e^–^+*n*H^+^⇌B, with A representing the proton acceptor, B the product, and *n* the number of electrons (protons). Examples include hydrogen evolution/oxidation reactions, oxygen reduction/evolution reactions, carbon dioxide reduction reaction etc. The Nernst equation expresses the equilibrium potential of these PCETs on the standard hydrogen electrode (SHE) scale *E*_eq_^SHE^ as, $E_{eq}^{SHE}=E_{eq}^{0,SHE}-2.3\frac{RT}{F}pH+\frac{RT}{nF}\ln\frac{a_{A}}{a_{B}}$ , with *E*_eq_^0,SHE^ being the standard equilibrium potential also referenced to the SHE, *a*_*i*_ the activity of species *i*. On the reversible hydrogen electrode (RHE) scale, $E_{eq}^{RHE}=E_{eq}^{0,SHE}+\frac{RT}{nF}\ln\frac{a_{A}}{a_{B}}$ is pH-independent. The polarization curves of several PCET reactions at different pHs are found to shift by around −60 mV/pH on the SHE scale, and they overlap on the RHE scale. Such Nernstian behaviors have been observed for hydrogen evolution/oxidation reactions (HER/HOR) on Au(111) and Ir-poly electrodes in acidic solutions^1^ and oxygen reduction reaction (ORR) on Pt(111) in solutions free of strongly adsorbing anions.^2,3^ When the polarization curves shift noticeably on the RHE scale, or by values significantly other than −60 mV/pH on the SHE scale, we are referring to non-Nernstian behaviors.

Non-Nernstian behaviors could be caused by multifaceted factors. On the one hand, when the reaction mechanism changes in solutions of different pHs, non-Nernstian behaviors are expected. This is the scenario for HER when the solution changes from the acidic to alkaline regime because the proton donor changes from protons to water.^1,4–7^ On the other hand, when the reaction kinetics is sluggish, the thermodynamic rationale behind the Nernstian behavior might be invalid, and a microkinetic analysis is often warranted. The kinetics of every PCET step is determined by the local reaction environment (LRE) in the electrical double layer (EDL), which are greatly influenced by the potential of zero charge (PZC).^8,9^ The PZC of electrode/electrolyte interfaces on the SHE scale is usually not sensitive to the change of solution pH.^10^ In other words, the PZC shifts by 60 mV/pH on the RHE scale. Therefore, even though the thermodynamic driving force of a PCET is invariant with the solution pH at a given electrode potential on the RHE scale, its kinetics changes since the LRE varies significantly. A prominent example for the latter scenario is H_2_O_2_ reduction/oxidation reactions at Pt(111) as studied by Feliu et al.^11,12^ Specifically, they observed an anomalously suppressed activity of H_2_O_2_ reduction at Pt(111) at low potentials. Moreover, the onset potential of this suppression shifts positively with the increase of solution pH on the RHE scale. These non-Nernstian behaviors have been recently rationalized by taking into account the pH-dependent surface charging behaviors and the multifaceted surface charge effects on the PCET steps.^13^ A comprehensive review of the non-Nernstian behaviors in capacitive and faradaic processes can be found in the work of Kastlunger et al.^14,15^

In this work, we investigate the pH effects on the ORR, the cathodic reaction of hydrogen–oxygen fuel cells that convert the chemical energy stored in hydrogen molecules to electricity.^16–18^ A previous work by some of us reported Nernstian behaviors in the ORR at Pt(111) in HClO_4_-based solutions.^2^ Reconciled with our results, Feliu et al. reported that the ORR at Pt(111) in HClO_4_-based solutions conforms to the Nernstian behaviors.^3^ Furthermore, they extended the conclusion to several stepped Pt single crystals with a varying proportion of step sites.^3^ In contrast, non-Nernstian behaviors have also been observed in ORR in the presence of specifically adsorbing anions.^19^ Introduction of bromide anions in the HClO_4_-based solutions leads to non-Nernstian behaviors in the ORR activity at Pt(111).^19^ This is rationalized by the pH-dependent adsorption energy of bromide on the RHE scale based on the observation that the onset of ORR coincides the potential of well-ordered bromide adsorption structure.^19^

Herein, we investigate the distinct pH effects on the ORR at the Pt(111)–acidic aqueous solution interfaces, with and without specifically adsorbing anions, and examine the influence of cations on anion-dependent deviations from Nernstian behavior. Specifically, ORR in HClO_4_/LiClO_4_ solutions follows a Nernstian shift, whereas H_2_SO_4_/Li_2_SO_4_ and H_2_SO_4_/Cs_2_SO_4_ solutions exhibit non-Nernstian behavior, with stronger deviations observed in Li^+^-containing solutions than in Cs^+^-containing ones. To elucidate these anion- and cation-dependent pH effects, we employ a systematic, hierarchical approach, progressively incorporating different levels of complexity. Our analysis begins with pure thermodynamic considerations, extends to intrinsic microkinetics, and ultimately considers LRE effects. To achieve this, we develop a multiscale theoretical framework that combines density functional theory (DFT) calculations of reaction mechanisms, a microkinetic model capturing multistep reaction dynamics, and a transport model incorporating EDL effects at the nanometer scale. Our findings reveal that adsorbed sulfate anions play a crucial proton-relaying role, fundamentally altering proton transfer pathways and leading to the observed non-Nernstian behavior in H_2_SO_4_-containing solutions. This insight underscores the importance of LRE modulation in electrocatalysis, providing a deeper understanding of electrolyte effects at the metal–solution interfaces.

## Anion- and Cation-Dependent pH Effects

The four-electron ORR in acidic electrolyte solutions consumes four protons for each oxygen molecule and forms two water molecules

1 $$O_{2}+4H^{+}+4e^{-}⇌2H_{2}O$$

where the electrolyte anion and cation play an implicit role. We used the Pt(111)­aqueous solution interfaces as model systems to examine the role(s) of anions and cations. Experimental details are provided in the methods section. Specifically, we compared two anions, ClO_4_^–^ and SO_4_^2–^, and two cations, Li^+^ and Cs^+^, in the study on the pH effects on ORR, see Figure 1. These two anions were chosen in this study for the following reasons. First, they represent two classes of anion adsorption at Pt(111): nonspecific adsorption for ClO_4_^–^ and specific adsorption for SO_4_^2–^.^20,21^ Second, the atomic structure of adsorbed SO_4_^2–^ on Pt(111) is well-known,^22–27^ allowing credible DFT calculations of the atomic reaction mechanism. Third, experimental data of both anions have been reported from several independent sources,^2,3,28,29^ allowing for an interlaboratory check of the experimental phenomena. Alkali metal cations have been widely used to understand the influence of cations on electrocatalytic reactions.^30–39^ Compared to Li^+^, Cs^+^ has a larger ionic radii but smaller hydrated radii with looser hydration shells,^38–40^ which could even specifically adsorb on highly negatively charged surfaces.^41^

**Figure 1:**
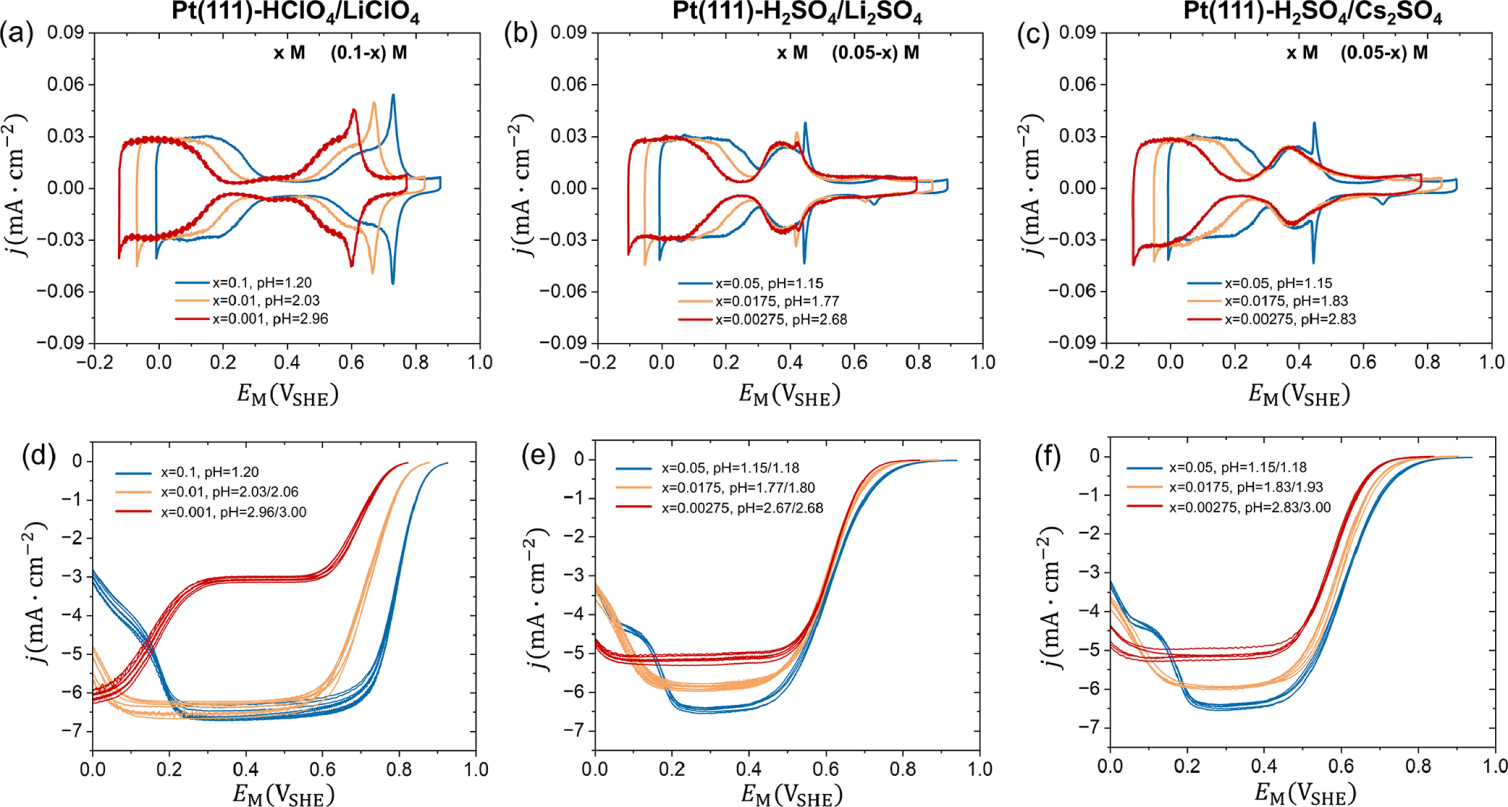
Cyclic voltammograms and ORR polarization curves at Pt(111) in (a, d) *x* M HClO_4_+(0.1–*x*) M LiClO_4_ (*x*=0.1, 0.01, 0.001), (b, e) *x* M H_2_SO_4_+(0.05–*x*) M Li_2_SO_4_ (*x*=0.05, 0.0175, 0.00275) and (c, f) *x* M H_2_SO_4_+(0.05–*x*) M Cs_2_SO_4_ (*x*=0.05, 0.0175, 0.00275) electrolyte solutions from pH 1 to 3. The electrode potential *E*_M_ applied on the working electrode is represented on the SHE scale. Each experiment was repeated at least five times. The sweep rate is 50 mV/s. The ORR activity was measured using the HMRDE configuration with a rotating rate of 2500 rpm. More details of the experiments are provided in the methods section and Section S1 of Supporting Information.

The cyclic voltammograms (CVs) of Pt(111) in the HClO_4_/LiClO_4_, H_2_SO_4_/Li_2_SO_4_ and H_2_SO_4_/Cs_2_SO_4_ electrolyte solutions as pH changes from 1 to 3 on the SHE scale are shown in Figure 1a–c. The same data on the RHE scale are provided in Figure S1. The comparison with the CVs reported in the literature in 0.1 M HClO_4_ ^42–45^ and 0.05 M H_2_SO_4_ solutions^28,46,47^ are shown in Figure S2, confirming that our Pt(111) electrode is well ordered and the cell used in the study is sufficiently clean. A typical hydrogen adsorption/desorption region between 0.05 V_RHE_ and 0.38 V_RHE_ is observed on Pt(111).^48^ For the HClO_4_/LiClO_4_ solutions, the nearly flat, low-lying region from 0.38 V_RHE_ to 0.6 V_RHE_ is assumed to be a double layer charging region,^49^ while some extent of chemisorption on defects cannot be excluded.^50^ The butterfly peak between 0.6 V_RHE_ and 0.9 V_RHE_ is attributed to hydroxyl adsorption/desorption.^49,51–53^ For the H_2_SO_4_/Li_2_SO_4_ and H_2_SO_4_/Cs_2_SO_4_ solutions, the sulfate adsorption process begins around 0.3 V_SHE_. It is nearly pH-independent in the potential range from 0.3 V_SHE_ to 0.5 V_SHE_, exhibiting a broad peak followed by a sharp spike. The sharp spike weakens or disappears in the solutions with Cs_2_SO_4_, as observed in the literature.^54^ This spike is considered to be originated from an order/disorder transition of adsorbed sulfate anions.^26,55–57^ In the potential range from 0.5 V_SHE_ to 0.8 V_SHE_, the sulfates remain adsorbed on the surface in the ordered structure as confirmed by electrochemical scanning tunneling microscopy (STM)^22^ and in situ surface X-ray scattering (SXS).^24^ The hydroxyl adsorption/desorption process is exceedingly inhibited, resulting in a slight peak from 0.7 V_RHE_ to 0.8 V_RHE_.

On the SHE scale, the polarization curves of ORR at Pt(111) in all solutions measured using the hanging-meniscus rotating disk electrode (HMRDE) configuration are shown in Figure 1d–f. The same set of data on the RHE scale is provided in Figure S1. Each experiment was repeated at least five times to average out errors caused by possible small contamination during experiments. The ORR current increases when the electrode potential *E*_M_ shifts from the onset potential to the negative direction before reaching the diffusion limiting current. For the case of the HClO_4_/LiClO_4_ solution at pH 3, shown in the red curves in Figure 1d, the first diffusion limiting current in the potential range from 0.3 V_SHE_ to 0.6 V_SHE_ is ascribed to proton diffusion and the second one below 0.05 V_SHE_ can be attributed to oxygen diffusion. It is interesting to note that the diffusion limiting current of oxygen changes with solution pH in SO_4_^2–^ solutions, while it is almost pH-independent in ClO_4_^–^ solutions.

Focusing on the kinetic region, we displayed in Figure 2 the electrode potentials on the SHE scale at two small ORR current densities −0.1 and −1 mA cm^–2^ as a function of the measured solution pH. The error bars are obtained from statistical analysis of five repeated experiments. The Nernst equation gives a slope of around −60 mV/pH for PCETs. The slopes in the HClO_4_/LiClO_4_ solutions are −59 mV/pH and −67 mV/pH for ORR current densities of −0.1 and −1 mA cm^–2^, respectively, which are very close to the Nernstian expectation. On the contrary, the slope in the H_2_SO_4_/Li_2_SO_4_ solutions at −0.1 mA cm^–2^ is −42 mV/pH. Surprisingly, the slope in the H_2_SO_4_/Li_2_SO_4_ solutions at −1 mA cm^–2^ is as low as −16 mV/pH, which differs with the Nernstian value by more than 40 mV/pH. We take this marked divergence as a strong signal of non-Nernstian behaviors for the ORR in H_2_SO_4_/Li_2_SO_4_ solutions. In other words, the polarization curves in the kinetic region almost overlap on the SHE scale for the ORR in H_2_SO_4_/Li_2_SO_4_ solutions. In H_2_SO_4_/Cs_2_SO_4_ solutions, we observe weaker non-Nernstian behaviors, with the slopes of −50 mV/pH and −36 mV/pH for ORR current densities of −0.1 and −1 mA cm^–2^, respectively. It is worth noting that the non-Nernstian behaviors are more significant at higher current densities.

**Figure 2:**
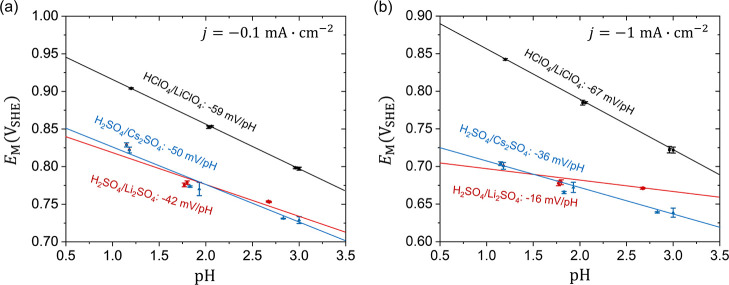
Relationship between the electrode potential and solution pH at ORR current density of (a) −0.1 mA cm^–2^ and (b) −1 mA cm^–2^ with the slopes marked. The data are taken from Figure 1d–f in the HClO_4_/LiClO_4_ (black, square), H_2_SO_4_/Li_2_SO_4_ (red, circle) and H_2_SO_4_/Cs_2_SO_4_ (blue, triangle) electrolyte solutions.

The origins of the distinct pH effects of ORR at Pt(111) in HClO_4_- and H_2_SO_4_-based solutions are dissected in a step-by-step manner. First, we examined the basic thermodynamics of ORR in both electrolyte solutions using DFT calculations, which is revealed to be insufficient to explain experimental observations. Second, we complemented DFT-calculated thermodynamics with intrinsic microkinetics, which explains a larger part of experimental observations. Third, we further added EDL effects, accounting for the different LRE in the presence of different electrolyte solutions, to quantitatively understand the experimental phenomena. The multifaceted analysis leads us to uncover the importance of the proton-relaying role of adsorbed sulfate in the non-Nernstian behaviors. Invoking this proton-relaying mechanism, a hierarchical model integrating DFT-calculated thermodynamics, multistep microkinetics, and the EDL effects provides a unified interpretation framework for the anion- and cation-dependent pH effects.

## Thermodynamic Analysis

We employed the computational hydrogen electrode (CHE) method^58,59^ to study the PCET reactions in ORR at Pt(111) in both absence and presence of specifically adsorbing anions. Technical details of DFT calculations are provided in the methods section and Section S2 of the Supporting Information. Most CHE-based DFT studies of ORR have been focused on the influence of the electrode structure, including various pure metals,^58,60^ different facets of these metals,^61^ metal alloys,^60,61^ and recently single atom catalysts.^62^ DFT-computed Pourbaix diagrams of anion adsorption on Pt(111) exist thanks to Groß et al. and others.^27,63,64^ However, to the best of our knowledge, the DFT study about the influence of sulfate anions on the ORR at Pt(111) is missing. Therefore, our DFT calculations presented below not only provide the atomistic basis for understanding distinct pH effects on ORR in HClO_4_ and H_2_SO_4_ solutions but also fill in a long-awaited missing piece of computational studies on ORR.

The calculated Gibbs free energy profile of ORR at Pt(111) in both solutions are shown in Figure 3. The initial structure of the Pt(111)–HClO_4_ aqueous interface is modeled as a four-layered 3×3 Pt slab covered by six ice-like water molecules, see the first subfigure of Figure 3a. The perchlorate anion is not explicitly considered in the model because it is revealed to be a weakly adsorbing anion.^29^ We note that more recent *ab initio* molecular dynamics (AIMD) simulations of water structures on Pt(111) suggest that the ice-like structure breaks down at room temperature and water molecules in the first layer form a dynamic mixture of 5-, 6-, and 7-membered rings.^65,66^ Considering that AIMD studies of the ORR is far from being mature and that only the first step is studied in a recent work,^67^ we used static DFT calculations with the hexagonal structure for water molecules. This also allows our results to be compared with the literature results wherean ice-like water layer was used to explicitly examine the roles of water molecules in the ORR.^68,69^

**Figure 3:**
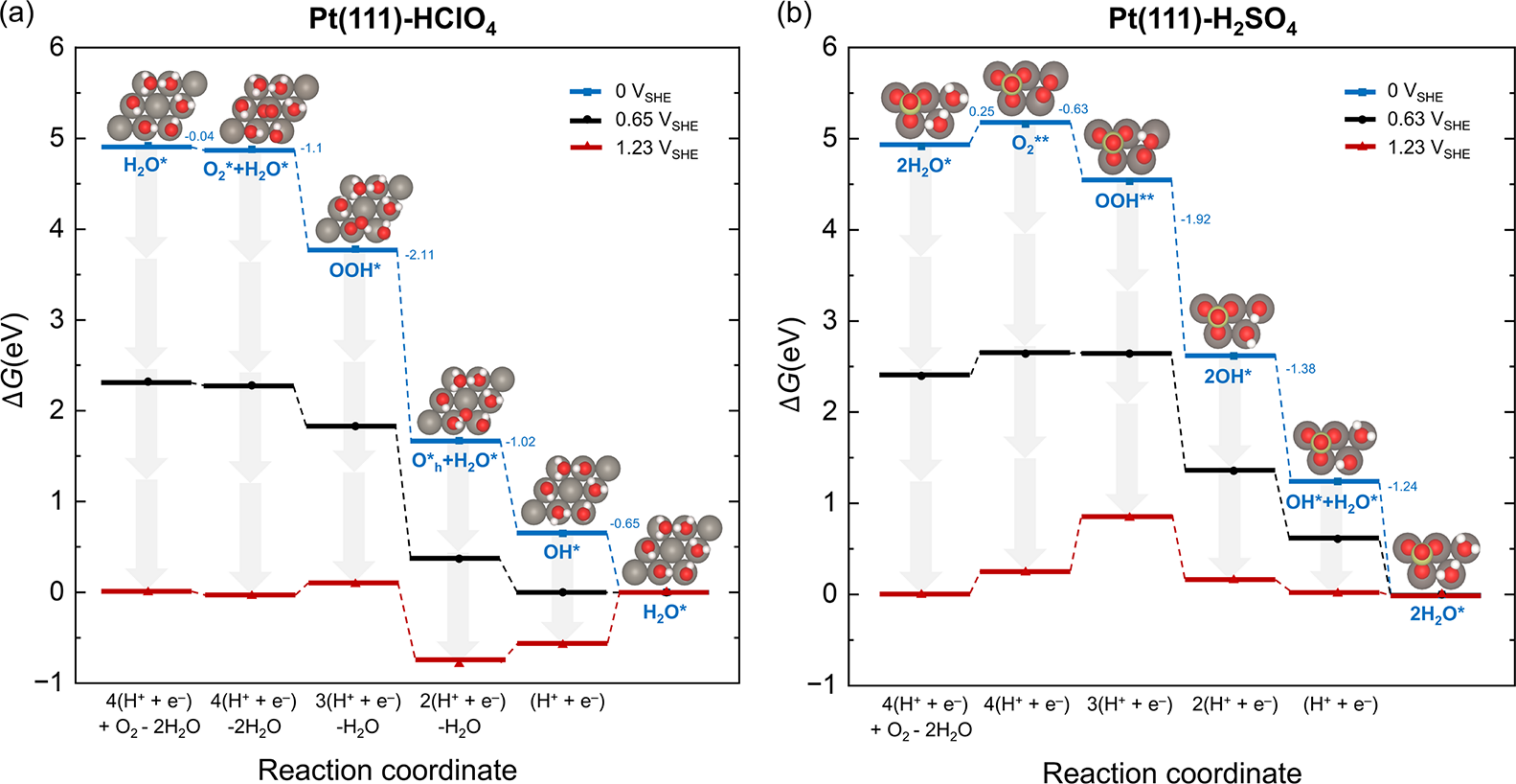
Gibbs free energy profile of the ORR under standard conditions at the (a) Pt(111)–HClO_4_ interface and (b) Pt(111)–H_2_SO_4_ interface at 0 V_SHE_ (blue square), 1.23 V_SHE_ (red triangle, the standard equilibrium potential), and 0.65/0.63 V_SHE_ (black circle, the minimum potential to keep all PCET steps exothermic) calculated using the CHE method. Alongside the free energy profile are the most stable static periodic unit cells (left: 3×3; right: $\sqrt{3}$ × $\sqrt{7}$ ) with one Pt atom layer and one adsorption layer, where Pt is gray, O red, H white, S yellow, generated using the VESTA software.^82^ The adsorbed species during the ORR are marked, while other reactants and products are listed on the *x*-axis. The standard Gibbs free energies of all steps at 0 V_SHE_ are given. More details of the DFT calculation can be found in the methods section and Section S2 of the Supporting Information.

STM,^22,55,70^ DFT calculations,^23^ in situ SXS,^24^ and CV results^25^ have suggested that adsorbed sulfate on Pt(111) forms a ( $\sqrt{3}$ × $\sqrt{7}$ )-R19.1° superstructure with a coverage of 0.2 monolayer, although the high-density (3×1) structure was also observed by STM.^22^ It has long been debated whether the adsorbed anion is SO_4_^2–^, SO_4_^2–^·H_3_O^+^ pair or HSO_4_^–^.^23,26,27,71–73^ A recent study, employing interface-specific vibrational sum frequency (VSF) spectroscopy with isotope exchange, suggests that SO_4_^2–^ is the dominant adsorbate for 0.5 M H_2_SO_4_.^26^ The phase diagram obtained by a grand-canonical DFT approach suggests that the adsorption of SO_4_^2–^ is more stable from pH 0 to 5 above 0.4 V_SHE_.^27^ A DFT study reported SO_4_^2–^·H_3_O^+^ pairs, and an adlayer of bisulfate with two water molecules at Pt(111)–H_2_SO_4_ interface.^23^ The estimated electrosorption valency suggests that there could be bisulfate adsorption before the onset of sulfate adsorption.^73^ In situ Fourier transform infrared (FTIR) spectroscopy suggests SO_4_^2–^·H_3_O^+^ pairs at Pt(111) in mixtures of H_2_SO_4_ and KOH solutions at pH 1.2, 2.0, 3.4.^71^ HSO_4_^–^ and the interconversion of HSO_4_^–^/H_3_O^+^ and H_2_SO_4_ were captured by infrared reflection absorption spectroscopy (IRAS) on Pt(111) in a 0.5 M H_2_SO_4_ solution.^72^ Considering the literature insights, we used SO_4_^2–^ as the adsorbed species as the starting point to explore the reaction mechanism of ORR at Pt(111)–H_2_SO_4_ interface. We will consider the case with HSO_4_^–^ at a later stage. In addition to the sulfate adsorbates, two water molecules likely adsorb on the remaining sites.^27^ The Gibbs free energy for the adsorption of the first water molecule is -0.61 eV, and that for the second is -0.49 eV, in agreement with the results reported by Gossenberger et al.^27^ To summarize, we modeled the Pt(111)–H_2_SO_4_ interface as a four-layer slab with the five surface Pt atoms occupied by one sulfate and two water molecules, shown in the first subfigure of Figure 3b.

A comprehensive comparison of different reaction pathways is conducted to determine the thermodynamically most favorable one, see Section S2 in the Supporting Information. The Gibbs free energies of elementary steps are obtained from the DFT-computed internal energy with thermal corrections, which are detailed in Tables S1–S5. Upon approaching the Pt(111)–HClO_4_ interface, one oxygen molecule will adsorb on the bare center site surrounded by six adsorbed water molecules, see the second subfigure of Figure 3a

2-1 $$O_{2}+*⇌O_{2}*\;\left(\Delta G_{1}^{0}=-0.04\;eV\right)$$

followed by

2-2 $$O_{2}*+H^{+}+e^{-}+H_{2}O*⇌OOH*+H_{2}O+*,\left(\Delta G_{2}^{0}=-1.1\;eV\right)$$

where * represents a top site, Δ*G*_*i*_^0^ is the Gibbs free energy of the *i* ^th^ step under standard conditions at 0 V_SHE_. Under other electrode potentials referenced to the RHE, the electrochemical potential of each electron–proton pair changes as −*e*_0_ *E*_M_, as shown in the gray arrows in Figure 3. We excluded the reaction path involving an adsorbed oxygen molecule occupying two top sites,^68,69,74^ denoted as O_2_** as shown in Figure S3, because the following step of OOH* formation will be endothermic above 0.26 V_SHE_ as shown in Table S5. The O–O bond in OOH* is cleaved in the subsequent PCET, forming one O*_h_ on the hollow site (*_h_) and one adsorbed water molecule

2-3 $$OOH*+H^{+}+e^{-}⇌O{*}_{h}+H_{2}O*\;\left(\Delta G_{3}^{0}=-2.11\;eV\right)$$

The ensuing PCET converts O*_h_ to OH*, removing one adsorbed water molecule

2-4 $$O{*}_{h}+H_{2}O*+H^{+}+e^{-}⇌OH*+H_{2}O\;\left(\Delta G_{4}^{0}=-1.02\;eV\right)$$

In the last step, the adsorbed OH* reacts with one proton and one electron

2-5 $$OH*+H^{+}+e^{-}⇌H_{2}O*\left(\Delta G_{5}^{0}=-0.65\;eV\right)$$

Our results agree decently with the literature results,^58,68,69,74–78^ see a quantitative comparison with two representative studies in Table 1 and more studies in Table S6. The differences are acceptable in view of normal DFT errors (±0.2 eV). The discrepancies between 13 studies including ours could be attributed to different adsorption configurations, water structure, OH* coverages, or functionals considered in these studies. The accuracy of the energies of O_2_*, O_2_** and OOH* could be improved by applying gas-phase corrections.^79^

**Table 1:** Gibbs Free Energy (eV) Data of ORR at Pt(111)–HClO_4_ Interface under Standard Conditions at 0 V_SHE_, See a More Comprehensive Comparison in Table S6

source	O_2_* O_2_**	OOH*	O*_h_	OH*	exchange-correlation functional	Pt size	solvent	Adsorbate coverage
Hansen et al., 2014 ^68^	4.99 (O_2_*)	3.91	1.7	0.75	RPBE	3×(3×2√3 )	one layer of explicit water	1/3 OH* to 1/3 O*
Liu et al., 2016 ^69^	4.37 (O_2_**)	3.87	1.50	0.67	PW91	4×(3×3)	Bilayer of explicit water	2/3 (OX*+H_2_O*)
this work	4.88 (O_2_*) 4.04 (O_2_**)	3.78	1.67	0.65	RPBE	4×(3×3)	one layer of explicit water	2/3 (OX*+H_2_O*)

For the ORR at the Pt(111)–H_2_SO_4_ interface, the thermodynamically most favorable pathway is obtained after a comprehensive comparison of various possible reaction pathways listed in Table S5, with the corresponding Gibbs energy profile shown in Figure 3b. Different from the case at the Pt(111)–HClO_4_ interface, the oxygen adsorption step is an endothermic step

3-1 $$O_{2}+2H_{2}O*⇌O_{2}**+2H_{2}O\;\left(\Delta G_{1}^{0}=0.25\;eV\right)$$

The adsorbed oxygen molecule will react with one proton and one electron to form an OOH**

3-2 $$O_{2}**+H^{+}+e^{-}⇌OOH**\;\left(\Delta G_{2}^{0}=-0.63\;eV\right)$$

The oxygen dissociation step is excluded here, as our computational test shows that placing two oxygen atoms in the slab always ends up with an oxygen molecule. Hereafter, three ways to transform OOH** are compared, see Table S5. It is most likely to form two OH* in the next step, expressed as

3-3 $$OOH**+H^{+}+e^{-}⇌2OH*\;\left(\Delta G_{3}^{0}=-1.92\;eV\right)$$

Afterward, two OH* would be reduced to two H_2_O*, successively

3-4 $$2OH*+H^{+}+e^{-}⇌OH*+H_{2}O*\;\left(\Delta G_{4}^{0}=-1.38\;eV\right)$$

3-5 $$OH*+H_{2}O*+H^{+}+e^{-}⇌2H_{2}O*\;\left(\Delta G_{5}^{0}=-1.24\;eV\right)$$

Three major differences between the DFT-calculated ORR mechanism in two electrolyte solutions have emerged. First, the local environment surrounding the active sites change from connected water molecules in HClO_4_ to a sulfate-water mixture in H_2_SO_4_ solution. Accompanying the structural shift from a 3×3 to a $\sqrt{3}$ × $\sqrt{7}$ Pt slab, the coverage of active sites for ORR changes from 2/3 to 2/5. Second, there is a change in the reaction pathway. The oxygen adsorption step is exothermic in HClO_4_ but endothermic in H_2_SO_4_ solution. Adsorbed OOH is transformed to O*_h_+H_2_O* in HClO_4_ and 2OH* in H_2_SO_4_ solution, which is caused by a change in the hydrogen bond network. Third, the potential-determining step (PDS) is different.^80^ The PDS is the desorption of OH* in the HClO_4_ solution, while it changes to the formation of OOH** in the H_2_SO_4_ solution. Our calculation results are in good agreement with the study on the dependence of ORR reaction mechanisms on the adsorption energy.^81^ Since the PDS in both cases is a PCET, the non-Nernstian behavior in the H_2_SO_4_ solution cannot be rationalized from the thermodynamic point of view. Consequently, a proper treatment of microkinetic and LRE is needed, which is the task of the next section.

## Intrinsic Microkinetic Analysis

The non-Nernstian behaviors in the H_2_SO_4_ based solutions cannot be understood from the foregoing DFT-based thermodynamics since the PDS is a PCET and Nernstian behaviors are expected. We conducted a microkinetic analysis to verify whether a rate-determining step (RDS) involving no proton could induce non-Nernstian behaviors.^80^ In the following analysis, a generalized concept, the rate determining resistance term (RDRT), is used, which incorporates the detailed kinetics and thermodynamics of multistep electrocatalytic reactions.^83^ The RDRT is defined as the maximum term of the overall reaction resistance, with detailed derivation and expressions in the method section, expressed as

4 $$RDRT\approx \max\left\{\frac{\Theta_{i}}{{\mathrm{k̅}}_{i}^{+}}\right\}$$

where Θ_*i*_ is a thermodynamic factor, read as

5 $$\Theta_{i}=\frac{1}{K_{i+1}K_{i+2}K_{i+3}K_{i+4}}+\frac{1}{K_{i+2}K_{i+3}K_{i+4}}+\frac{1}{K_{i+3}K_{i+4}}+\frac{1}{K_{i+4}}+1$$

with $K_{i}={\mathrm{k̅}}_{i}^{+}/{\mathrm{k̅}}_{i}^{-}$ being the equilibrium constant of the *i* ^th^ elementary step and *K*_*i*_ = *K*_*i*–5_ as *i* > 5. ${\mathrm{k̅}}_{i}^{\pm }$ is the forward (+) or backward (−) reaction rate constant of the *i* ^th^ elementary step considering the concentrations of all non-adsorbed reactants. For example, ${\mathrm{k̅}}_{1}^{+}=k_{1}^{+}{\mathrm{c̃}}_{O_{2}}$ is composed of a reaction rate constant *k*_1_^+^ and a dimensionless (tilde) oxygen concentration referenced to its standard value.

The intrinsic kinetic model neglects the EDL effects, namely, the concentrations and the electric potential at the interface are the same as these in the bulk solution. The bulk concentrations, ${\mathrm{c̃}}_{i}={\mathrm{c̃}}_{i}^{b}$ (b: bulk solution), are listed in Table S8, and the overpotential is calculated by *η*_*i*_ = *E*_M_ – *E*_*i*_^eq,0^, where $E_{i}^{eq,0}=-\frac{\Delta G_{i}^{0}}{e_{0}}$ is the standard equilibrium potential of *i* ^th^ elementary step calculated using Δ*G*_*i*_^0^ from Figure 3. The activation barrier is influenced by the LRE, including but not limited to surface charge, electronic interaction, solvation environment and bond strength.^84,85^ We found it is necessary to assume a pH-dependent activation barrier for PCET steps in order to reproduce −60 mV/pH in the kinetic region on the SHE scale. Otherwise, the polarization curves shift by around −120 mV/pH as shown in Figure S4. Specifically, we used a pH-dependent reorganization energy, in the linear approximation, as $\lambda =\lambda_{0}+\frac{\partial \lambda }{\partial pH}\cdot pH$ where *λ*_0_ is the reorganization energy at pH 0 and $\frac{\partial \lambda }{\partial pH}$ is fitted as −0.12 eV for all solutions. To avoid overparameterization, a single solvent reorganization free energy is used for all elementary steps in one solution, that is, *λ*_*i*_ = *λ*. More details of the intrinsic kinetic model are provided in the methods section and Section S3 of the Supporting Information.

Nernstian behavior is captured by the intrinsic kinetic model in the HClO_4_/LiClO_4_ solutions as shown in Figure 4a, which can be understood using the RDRT as an analytical tool. The resistive terms of $\frac{\Theta_{i}}{{\mathrm{k̅}}_{i}^{+}}$ in 0.1 M HClO_4_ solution are shown in Figure 4b. $\frac{\Theta_{2}}{{\mathrm{k̅}}_{2}^{+}}$ is the RDRT in the potential region positive of 0.8 V_SHE_. The dominating term in Θ_2_ is ${\left(K_{4}K_{5}K_{1}\right)}^{-1}+{\left(K_{5}K_{1}\right)}^{-1}$ since *K*_5_<*K*_1_,1,*K*_4_≪*K*_3_. The RDRT changes from ${\left(K_{4}K_{5}K_{1}{\mathrm{k̅}}_{2}^{+}\right)}^{-1}$ to ${\left(K_{5}K_{1}{\mathrm{k̅}}_{2}^{+}\right)}^{-1}$ when the potential is negative of 0.95 V_SHE_ as shown in the dotted and dashed lines. In the potential region between 0.8 to 0.95 V_SHE_, the overall current of ORR is proportional to $K_{5}K_{1}{\mathrm{k̅}}_{2}^{+}$ , namely, $j_{ORR}\propto \frac{k_{5}^{+}{\mathrm{c̃}}_{H^{+}}^{b}}{k_{5}^{-}}\frac{k_{1}^{+}{\mathrm{c̃}}_{O_{2}}^{b}}{k_{1}^{-}}k_{2}^{+}{\mathrm{c̃}}_{H^{+}}^{b}$ . The first term *K*_5_, the equilibrium constant of a PCET step expressed in eq [Disp-formula eq2-5], obeys the Nernstian behavior. *K*_1_ is the equilibrium constant of oxygen adsorption step as shown in eq [Disp-formula eq2-1], which is independent of ${\mathrm{c̃}}_{H^{+}}^{b}$ and *E*_M_. $k_{2}^{+}{\mathrm{c̃}}_{H^{+}}^{b}$ , corresponding to the PCET step eq [Disp-formula eq2-2], also obeys the Nernstian behavior with the pH-dependent activation barrier.^84,85^ The RDRTs for other HClO_4_/LiClO_4_ solutions, as shown in Figure S5, can be analyzed in a similar manner. It is noted that the calculated Tafel slope is smaller than the experiment data, due to the neglect of EDL effects to be considered in the next section.

**Figure 4:**
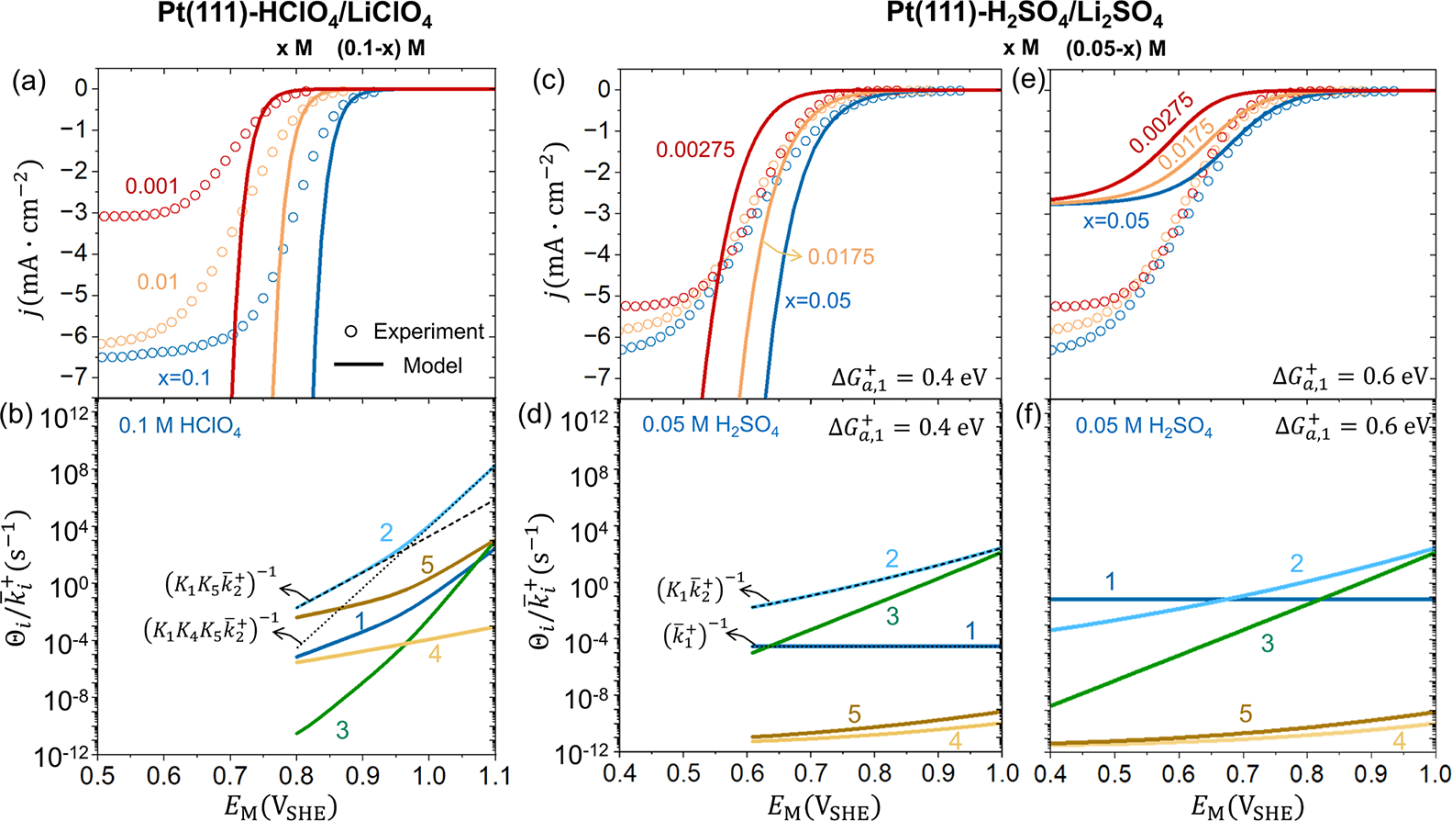
ORR polarization curves obtained by experiment (circles) and intrinsic kinetic model (curves) at Pt(111) in (a) *x* M HClO_4_+(0.1–*x*) M LiClO_4_ (*x* = 0.1, 0.01, 0.001) and (c, e) *x* M H_2_SO_4_ + (0.05–*x*) M Li_2_SO_4_ (*x* = 0.05, 0.0175, 0.00275) solutions. The resistive terms of $\frac{\Theta_{i}}{{\mathrm{k̅}}_{i}^{+}}$ in 0.1 M HClO_4_ and 0.05 M H_2_SO_4_ solutions are shown in (b, d, f). Two values of the activation energy of the oxygen adsorption step Δ*G*_a,1_^+^ are used for H_2_SO_4_/Li_2_SO_4_ solutions: (c, d) 0.4 eV and (e, f) 0.6 eV. More details of the intrinsic kinetic model are provided in the methods section and Section S3 of the Supporting Information.

The intrinsic kinetic model predicts Nernstian behaviors also for the H_2_SO_4_/Li_2_SO_4_ solution as shown in Figure 4c, contrasting the experimental phenomena. The RDRT in 0.05 M H_2_SO_4_ solution is $\frac{\Theta_{2}}{{\mathrm{k̅}}_{2}^{+}}$ in the potential region between 0.6 to 1.0 V_SHE_ as shown in Figure 4d. $\frac{\Theta_{2}}{{\mathrm{k̅}}_{2}^{+}}$ is approximated as $\frac{1}{K_{1}{\mathrm{k̅}}_{2}^{+}}$ since *K*_1_<1≪*K*_5_<*K*_4_≪*K*_3_. Therefore, the overall current density is proportional to $K_{1}{\mathrm{k̅}}_{2}^{+}$ , namely, $j_{ORR}\propto \frac{k_{1}^{+}{\mathrm{c̃}}_{O_{2}}^{b}}{k_{1}^{-}}k_{2}^{+}{\mathrm{c̃}}_{H^{+}}^{b}$ . Nernstian behavior is expected because *K*_1_ is independent of ${\mathrm{c̃}}_{H^{+}}^{b}$ and *E*_M_, and $k_{2}^{+}{\mathrm{c̃}}_{H^{+}}^{b}$ also obeys Nernstian behavior.^84,85^ The RDRTs for other H_2_SO_4_/Li_2_SO_4_ solutions, as shown in Figure S5, all lead to Nernstian behavior in H_2_SO_4_/Li_2_SO_4_ solutions.

We also tested the hypothesis that the non-Nernstian behavior is induced by a RDRT involving no proton. $\frac{\Theta_{1}}{{\mathrm{k̅}}_{1}^{+}}$ is the only one resistive term without ${\mathrm{c̃}}_{H^{+}}^{b}$ . Θ_1_ is approximately 1 since *K*_2_,1≪*K*_5_<*K*_4_≪*K*_3_. To make $\frac{1}{{\mathrm{k̅}}_{1}^{+}}$ the RDRT, we increase the activation energy of the oxygen adsorption step Δ*G*_a,1_^+^. As expected, $\frac{1}{{\mathrm{k̅}}_{1}^{+}}$ becomes the RDRT at potentials negative of 0.65 V_SHE_, as shown in Figure 4f. However, since $j_{ORR}\propto k_{1}^{+}{\mathrm{c̃}}_{O_{2}}^{b}$ in this scenario, the ORR current density becomes potential-independent and smaller as shown in Figure 4e, deviating from the experimental phenomena. In summary, the non-Nernstian behaviors cannot be rationalized by the intrinsic kinetic model.

## Anion Dependent Local Reaction Environment

The gap between experimental observations and preceding analysis triggers us to further consider the LRE determined by the EDL effects and mass transport. The LRE is important for electrocatalytic reactions because the local electric potential and concentrations are different from the bulk conditions, which was first realized by Frumkin.^86,87^ We have developed a theoretical framework to model the LRE based on modified Poisson-Nernst–Planck (PNP) equations that are coupled with multielectron reactions on the metal–solution interface.^9,13,35,83,88–103^ The details of the model are provided in the methods section.

The LRE is revealed to be anion-dependent, as schematically shown in Figure 5a,b. The LRE at Pt(111) in 0.1 M HClO_4_ and 0.05 M H_2_SO_4_ interfaces are compared in terms of the potential distributions at 0.8 V_SHE_, the surface free charge density, and the local proton concentration as shown in Figure 5. The closest solvated ions are located on the outer Helmholtz plane (OHP). The model features a detailed consideration of ionic adsorbates like sulfate anions and oxygen-containing species that are located on the inner Helmholtz plane (IHP) with partial charges.^104–107^ Thermodynamic analysis suggests that each adsorbed sulfate on Pt(111) has a net charge ranging from −2*e*_0_ to −1.3*e*_0_ as the electrode potential changes from 0.45 V_RHE_ to 0.65 V_RHE_ in solutions of H_2_SO_4_ with an excess of 0.1 M HClO_4_.^108^ The partially charged adsorbed sulfate anion induces a significant surface dipole, expressed as

6 $$\mu_{{\mathrm{SO}}_{4}}=e_{0}\zeta_{SO_{4}}\delta_{IHP}\theta_{SO_{4}}n_{M}$$

where *e*_0_ is the elementary charge, $\zeta_{SO_{4}}$ the electron number taken by each sulfate adsorbate, *δ*_IHP_ the distance from metal surface to the IHP, $\theta_{SO_{4}}$ the coverage of sulfate adsorbates, *n*_M_ the areal number density of metal atoms. We took $\zeta_{SO_{4}}=1$ and $\theta_{SO_{4}}=0.2$ in the potential region positive of 0.5 V_SHE_ where the sulfate adsorbates have an ordered structure as shown in Figure 3b according to previous studies.^22,24^ Focusing on the non-Nernstian behaviors positive of 0.5 V_SHE_, we have neglected the model results negative of 0.5 V_SHE_. Another reason for this neglect is that $\theta_{SO_{4}}$ varies with electrode potential negative of 0.5 V_SHE_, while our DFT calculations assume a full coverage of $\theta_{SO_{4}}$ .

**Figure 5:**
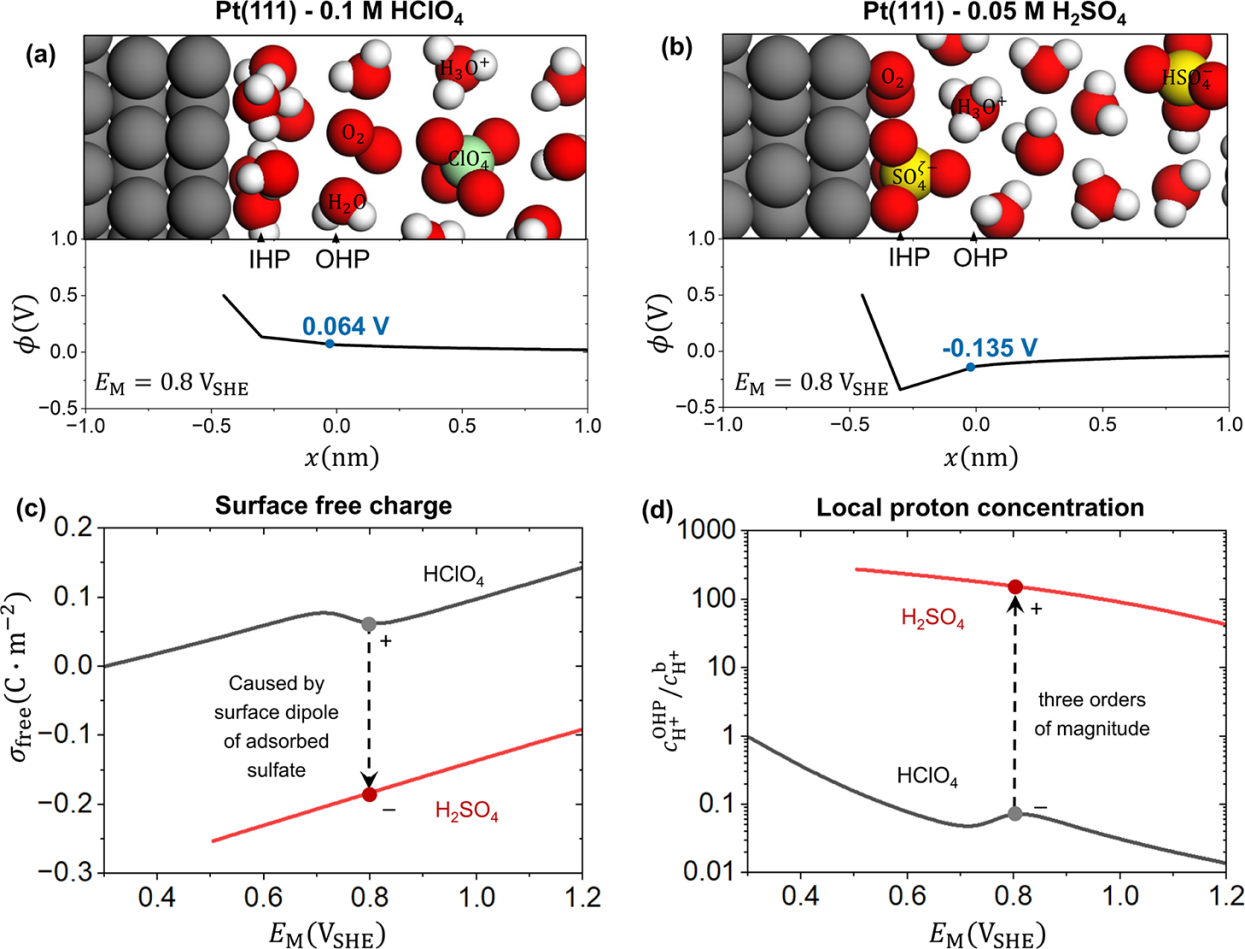
Schematic illustrations of (a) Pt(111)-0.1 M HClO_4_ and (b) Pt(111)-0.05 M H_2_SO_4_ interfaces along with the calculated distributions of the electric potential at 0.8 V_SHE_ from the metal surface to the electrolyte solution. Pt is gray, O red, H white, Cl green and S yellow. The electric potentials at the OHP are marked. The potential-dependent (c) surface free charge density and (d) proton concentration at the OHP in these two solutions are shown (black: 0.1 M HClO_4_, red: 0.05 M H_2_SO_4_). Model results in the H_2_SO_4_ solution negative of 0.5 V_SHE_ are not shown since the coverage and structure of adsorbed sulfate change significantly with electrode potential in this potential range while this potential dependency is not considered in our model focusing on the non-Nernstian behavior positive of 0.6 V_SHE_. For more details of the model, readers are referred to the methods section and the Section S4 of Supporting Information.

The surface dipole brings about an electric potential drop from the metal surface to the IHP, *μ*_SO4_/*ϵ*_IHP_, with *ϵ*_IHP_ being the dielectric permittivity of the space between the metal surface and the IHP. As a result, the electric potential in the solution phase becomes negative, as shown in Figure 5b, though the electrode potential is far positive of the PZC of the bare Pt(111).^10^ Accordingly, the surface free charge density, as shown in the red curve in Figure 5c, is negative due to the partially charged adsorbed sulfate anions in the potential region relevant to the ORR. Nonmonotonic surface charging behaviors are also observed in the HClO_4_ solution due to the adsorbed O and OH of which the coverages are shown in Figure S6.^88,100,109^ The negative excess free charge in H_2_SO_4_ solution attracts more protons in the EDL. In the potential relevant to the ORR, $c_{H^{+}}^{OHP}/c_{H^{+}}^{b}$ in 0.05 M H_2_SO_4_ solution is almost three orders of magnitude higher than that in 0.1 M HClO_4_ solution, as shown in Figure 5d. In summary, the surface free charge is greatly influenced by the partially charged adsorbed sulfate anion, drastically changing the LRE.

The hierarchical theoretical model considering the LRE quantitatively captures the polarization curves in HClO_4_/LiClO_4_ solutions, including both diffusion-limiting phenomena and Nernstian behaviors, as shown in Figure 6a. It is noted that we have adjusted the adsorption free energies within the common DFT error of 0.2 eV, as listed in Table S13, in bringing the model and experiments to a quantitative agreement. The adjustment in the adsorption free energies could be considered as a correction to DFT calculations where important LRE effects are not considered.^64,69,74,76,110^ Detailed microkinetic analysis in Figure 6b further reveals that the RDRT is $\frac{\Theta_{2}}{{\mathrm{k̅}}_{2}^{+}}$ in 0.1 M HClO_4_ solution from 0.3 V_SHE_ to 1.2 V_SHE_. The Tafel slope, defined as $b=-\frac{\partial E_{M}}{\partial \log\left|j_{ORR}\right|}$ , could be obtained by $b\propto \frac{\partial E_{M}}{\partial \log\frac{\Theta_{2}}{{\mathrm{k̅}}_{2}^{+}}}$ from Figure 6b. *b* increases continuously from around 24 mV/dec to 40 mV/dec than to infinite when the dominating term in Θ_2_ changes from ${\left(K_{4}K_{5}K_{1}\right)}^{-1}$ to ${\left(K_{5}K_{1}\right)}^{-1}$ then to ${\left(K_{1}\right)}^{-1}$ as *E*_M_ becomes more negative, which agrees with the previous analysis.^88,94^

**Figure 6:**
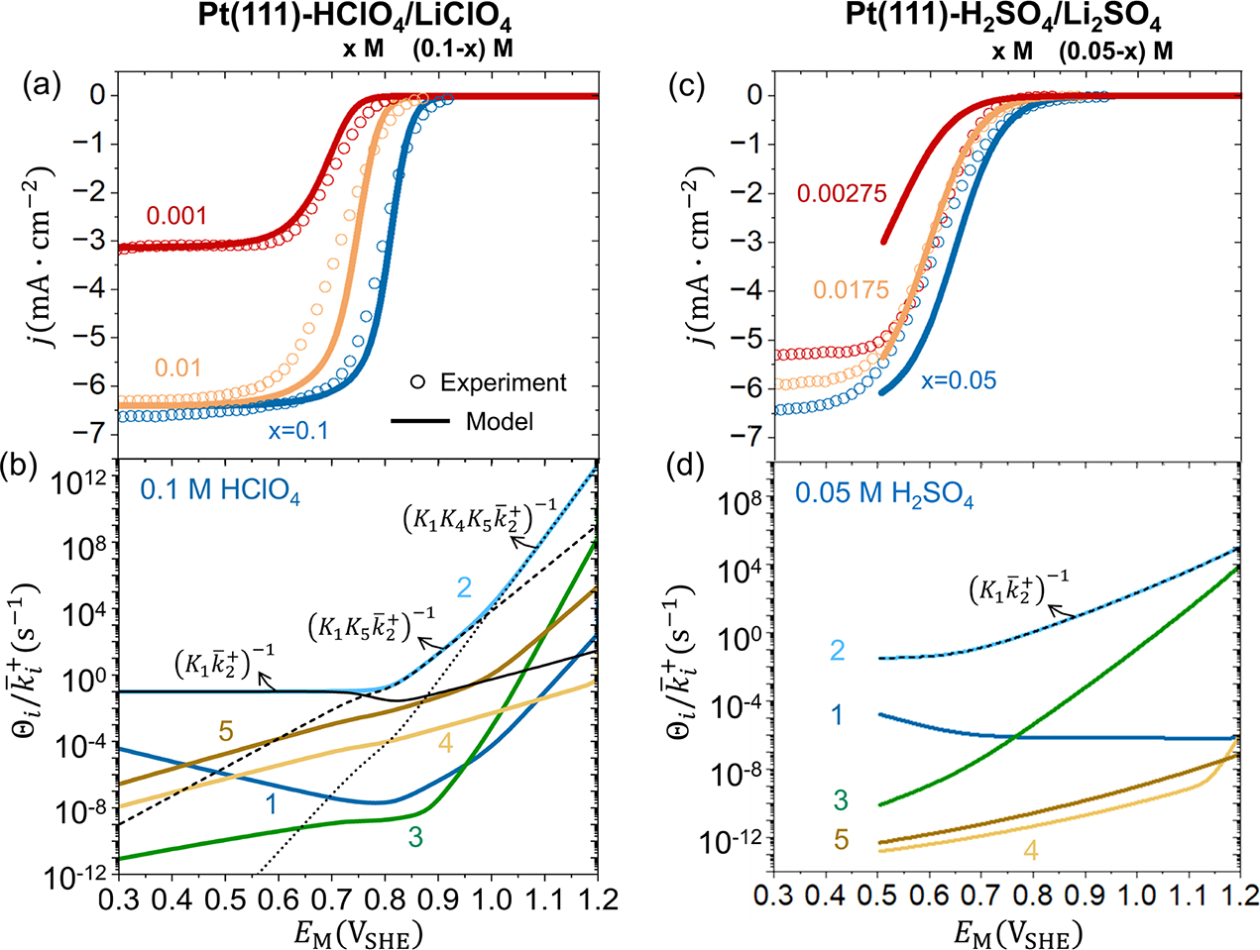
ORR polarization curves obtained in experiments (circles) and calculated by the hierarchical theoretical model (curves) at Pt(111) in (a) *x* M HClO_4_+(0.1–*x*) M LiClO_4_ (*x*=0.1, 0.01, 0.001) and (c) *x* M H_2_SO_4_+(0.05–*x*) M Li_2_SO_4_ (*x*=0.05, 0.0175, 0.00275) solutions. The resistive terms of $\frac{\Theta_{i}}{{\mathrm{k̅}}_{i}^{+}}$ in 0.1 M HClO_4_ and 0.05 M H_2_SO_4_ solutions are shown in (b,d). More details are provided in the methods section and Section S4 of the Supporting Information.

Similar to the results shown in Figure 5, the electric potential and surface free charge density are positive for HClO_4_/LiClO_4_ solutions at pH 2 and 3 as shown in Figure S7. The nonmonotonic surface charging behaviors occur at more negative potentials at higher pHs due to the earlier formation of OH* and O* as shown in Figure S6. Cations are repelled and anions are attracted by the positive surface free charge as shown in Figure S8. The current decreases compared to the intrinsic kinetic model results since ${\mathrm{c̃}}_{H^{+}}^{OHP}<{\mathrm{c̃}}_{H^{+}}^{b}$ although the driving force increases expressed by |*η*_*i*_| = −(*E*_M_ – *ϕ*_OHP_ −*E*_*i*_^eq,0^) with a positive *ϕ*_OHP_.^111^ The RDRT at pH 2 and 3 are similar to that at pH 1 as shown in Figure S9a. In the diffusion limiting region, the local oxygen concentration decreases to around 0 at pH 1 and 2, while the local proton concentration decreases to around 0 at pH 3 as shown in Figure S8a,c.

In sharp contrast with experiments, Nernstian behaviors are obtained from the hierarchical model for ORR in the H_2_SO_4_/Li_2_SO_4_ solutions as shown in Figure 6c. Detailed microkinetic analysis reveals that the RDRT from 0.5 V_SHE_ to 1.0 V_SHE_ is still $\frac{\Theta_{2}}{{\mathrm{k̅}}_{2}^{+}}\approx \frac{1}{K_{1}{\mathrm{k̅}}_{2}^{+}}$ in the H_2_SO_4_/Li_2_SO_4_ solutions from pH 1 to 3 as shown in Figures 6d and S9b. The current density is proportional to $K_{1}{\mathrm{k̅}}_{2}^{+}$ , namely, $j_{ORR}\propto \frac{k_{1}^{+}{\mathrm{c̃}}_{O_{2}}^{OHP}}{k_{1}^{-}}k_{2}^{+}{\mathrm{c̃}}_{H^{+}}^{OHP}$ . The surface free charges are all negative from pH 1 to 3 as shown in Figure S7, which attract cations and repel anions. ${\mathrm{c̃}}_{H^{+}}^{OHP}$ is much larger than ${\mathrm{c̃}}_{H^{+}}^{b}$ as shown in Figure S8, while the promotion degree as ${\mathrm{c̃}}_{H^{+}}^{OHP}/{\mathrm{c̃}}_{H^{+}}^{b}$ are almost same from pH 1 to 3. The current is improved by the higher proton concentration even though the driving force decreases, while the fitted *λ*_0_ is larger compared to that used in the intrinsic kinetic model as listed in Tables S9 and S13. The hierarchical model considering conventional EDL effects still cannot capture the non-Nernstian behaviors in H_2_SO_4_/Li_2_SO_4_ solutions.

## Proton-Relaying Role of Adsorbed Sulfate

To rationalize the non-Nernstian behaviors observed in Figure 1e-f, we hypothesize that protons, exceedingly accumulated in the OHP as shown in Figures 5d and S8d, are bonded with the adsorbed sulfate anion, expressed as

7 $${\left(SO_{4}^{\zeta -}\right)}_{ad}+H_{3}O^{+}⇌{\left(HSO_{4}^{\left(\zeta -1\right)-}\right)}_{ad}+H_{2}O$$

where “ad” means adsorbates. This hypothesis is reconciled with current understanding of the chemical nature of the adsorbed anion in H_2_SO_4_ solution; adsorbed states of SO_4_^2–^·H_3_O^+^ pair and HSO_4_^–^ have been reported in refs 23,71–73 . This hypothesis implies a change of the proton donor in the ORR from hydrated protons to adsorbed HSO_4_^(ζ–1)–^. In other words, the adsorbed sulfate behaves as the proton-relaying in the ORR in H_2_SO_4_/M_2_SO_4_ (M = Li, Cs) solutions.

We conducted further DFT calculations to validate the hypothesis. Specifically, we studied whether a proton near the surface prefers bonding with adsorbed SO_4_^ζ–^ or O_2_. The Gibbs free energy for the systems with one ${\left(HSO_{4}^{\left(\zeta -1\right)-}\right)}_{ad}$ is lower than the nonbonded states as shown in Figure 7a and more details in Figure S6. The energy difference is from 0.27 to 0.43 eV for different structures, in agreement with the results reported by Gossenberger et al.^27^ In one configuration of ${\left(HSO_{4}^{\left(\zeta -1\right)-}\right)}_{ad}$ with H pointing to the adsorbed O_2_, this adsorbed H prefers to form a bond with oxygen. To summarize, our DFT calculations support the likelihood of the hypothesis of the proton-relaying role for the adsorbed SO_4_^ζ–^ during the ORR, though we have not excluded the possibility of protons directly bonded to the oxygen molecule.

**Figure 7:**
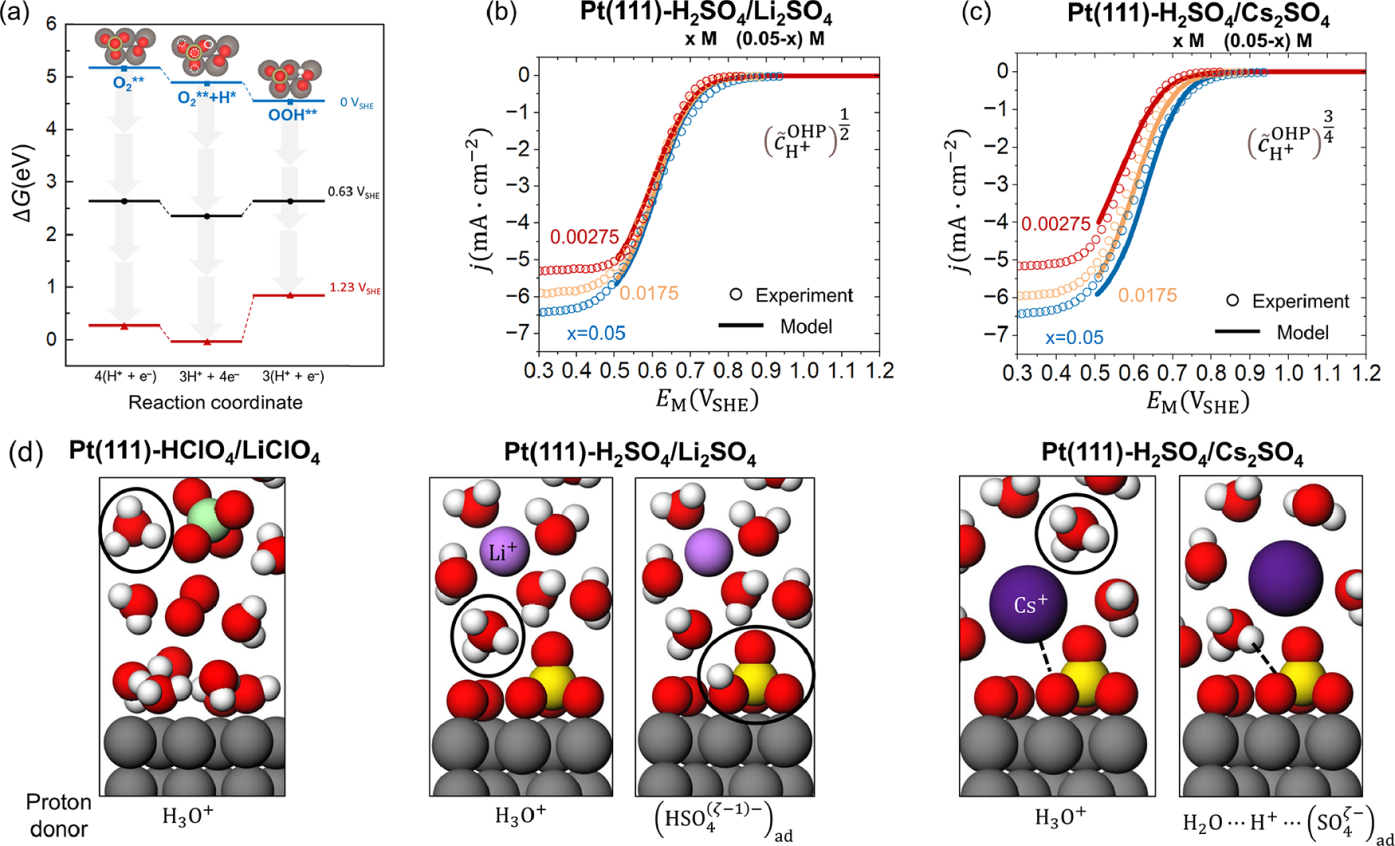
(a) The Gibbs free energy profile of the formation of OOH adsorbate at Pt(111)-H_2_SO_4_ interface at 0 V_SHE_ (blue square), 0.63 V_SHE_ (black circle) and 1.23 V_SHE_ (red triangle). Comparison of the polarization curves of the ORR (b) in H_2_SO_4_/Li_2_SO_4_ solutions and (c) H_2_SO_4_/Cs_2_SO_4_ solutions from pH 1 to 3. The circles represent the experiment data while the curves are the hierarchical theoretical model results with the reduced proton reaction order as 0.5 in (b) and 0.75 in (c). (d) Schematic diagram of the interface highlighting the proton-relaying role of adsorbed sulfate anions to understand the anion and cation dependent pH effects of ORR. Pt is gray, O red, H white, Cl green, S yellow, Li purple and Cs dark purple.

The hypothesis leads to a modification to the local proton concentration in the rate expressions in the microkinetic submodel. Specifically, incorporated in the rate constant is now ${\left({\mathrm{c̃}}_{H^{+}}^{OHP}\right)}^{\alpha }$ , with the exponent 0≤*α*≤ 1 quantifying the relative contributions of two proton donors: hydrated protons or ${\left(HSO_{4}^{\left(\zeta -1\right)-}\right)}_{ad}$ . *α*=1 represents the case where hydrated protons are the solo proton donor, while *α*=0 represents the case where ${\left(HSO_{4}^{\left(\zeta -1\right)-}\right)}_{ad}$ serve as the solo proton donor. 0<*α*<1 represents the general case where both proton donors participate in the ORR. Our modified hierarchical theoretical model now reproduces the non-Nernstian behaviors in H_2_SO_4_/M_2_SO_4_ (M = Li, Cs) solutions, as shown in Figure 7b,c. The fitting proton reaction order is 0.5 for the H_2_SO_4_/Li_2_SO_4_ solutions and 0.75 for the H_2_SO_4_/Cs_2_SO_4_ solutions. Cs^+^ has a weaker hydration shell compared to Li^+^. It is easier for Cs^+^ to be bound with adsorbed sulfate, which decreases the change to form ${\left(HSO_{4}^{\left(\zeta -1\right)-}\right)}_{ad}$ as described in Figure 7d. Therefore, *α* in H_2_SO_4_/Cs_2_SO_4_ solutions is larger than that in H_2_SO_4_/Li_2_SO_4_ solutions with the decreased extent of the non-Nernstian behavior.

## Conclusion

In this work, we investigated the pH-dependent behavior of the ORR at Pt(111) in different electrolyte environments, revealing significant deviations from Nernstian behavior in H_2_SO_4_-containing solutions. While ORR in HClO_4_/LiClO_4_ solutions follows a Nernstian shift from pH 1 to 3, H_2_SO_4_/Li_2_SO_4_ and H_2_SO_4_/Cs_2_SO_4_ solutions exhibit non-Nernstian behaviors, with stronger deviations observed in Li^+^-containing solutions compared to Cs^+^.

To interpret these findings, we developed a hierarchical theoretical framework for multielectron reactions at the metal–solution interfaces. This approach integrates DFT calculations, intrinsic microkinetic modeling, and a LRE model that accounts for mass transport and EDL effects. Our DFT calculations reveal that in H_2_SO_4_ solutions, the PDS is a PCET process, significantly influenced by the adsorption of sulfate anions.

Our analysis further demonstrates that adsorbed sulfate anions induce a surface dipole, leading to a negative surface free charge, which in turn attracts hydrated protons via Coulomb interactions. This results in the formation of adsorbed bisulfate species, which then serve as the proton donor in ORR along with the free hydrated proton. This shift in the proton donor species explains the observed reduction in proton reaction order from 1 in HClO_4_/LiClO_4_ solutions to 0.5 in H_2_SO_4_/Li_2_SO_4_ and 0.75 in H_2_SO_4_/Cs_2_SO_4_.

By establishing the role of anion- and cation-dependent LRE effects in electrocatalysis, this work provides a new mechanistic perspective on electrolyte modulation of reaction kinetics. Furthermore, it demonstrates how a combined theoretical and computational approach can disentangle complex, multiscale interactions at the electrochemical interface. These insights not only advance fundamental understanding of ORR mechanisms but also have broader implications for rational electrolyte design in electrocatalysis and energy conversion technologies.

## Methods

### Electrochemical Experiment

The platinum single-crystal electrode as working electrode (WE) was prepared following the Clavilier method.^112^ We melted the end of the platinum wire (with the diameter Φ = 0.5 mm) in the H_2_/O_2_ flame to form a platinum bead with the diameter of 2∼3 mm, then oriented using the diffraction spots from a laser, followed by cutting and polishing steps to expose the desired plane (111). Before immersed in the electrochemical cell, the platinum electrode is annealed by inductive heating at ∼380 A for 60 s. It was then completely cooled in a reductive atmosphere with Ar and quenched in ultrapure water saturated with Ar gas. Finally, it was shielded with a droplet of water to protect it from impurities in the air. The electrolyte solutions were prepared with perchloric acid (Aladdin, AR), sulfuric acid (Sigma-Aldrich), LiClO_4_ (Aladdin, 99.9% metals basis), Li_2_SO_4_ (Aladdin, 99.9% metals basis), and ultrapure water (18.2 MΩ cm, from Milli-Q water system). The electrolyte solutions are *x* M HClO_4_ + (0.1–*x*) M LiClO_4_ with *x* = 0.1, 0.01, 0.001, *x* M H_2_SO_4_ + (0.05–*x*) M Li_2_SO_4_ with *x* = 0.05, 0.0175, 0.00275 and *x* M H_2_SO_4_ + (0.05−*x*) M Cs_2_SO_4_ with *x* = 0.05, 0.0175, 0.00275. The solution pHs were measured by a pH meter (LiChen pH-100B) before electrochemistry experiments. All solutions were purged with Ar for 20 min before experiments, which is the Ar mixing O_2_ gas with the purity of 99.99% purchased from Nanjing Shang Yuan industrial gas company. Cyclic voltammetry experiments were carried out at room temperature (25 ± 1 °C) in a three-electrode all-glass cell, involving a Pt wire as the counter electrode and the Ag/AgCl electrode as the reference electrode. Our control experiment confirmed that the solutions are not contaminated by chloride within 5 h of measurements. The CVs were recorded under a scan rate of 50 mV/s. The electrode potential was controlled by a potentiostat (Autolab 302N). The polarization curves of the oxygen reduction reaction were measured on the Pt(111) electrode in the O_2_ saturated solutions with the potential scan rate as 50 mV/s and rotation speed of 2500 rpm under the hanging-meniscus rotating disk electrode (HMRDE). The electrode rotating speed was controlled by a modulated rotator (Hokuto Denko Ltd.). The electrolyte solutions were purged continuously with Ar during the experiment process. 90% ohm compensation were performed.^113^ Current densities are normalized by the geometric surface area of Pt(111) as 0.038 cm^2^. All experiments were repeated at least five times.

### DFT Calculation

All electronic structure calculations were carried out using the Vienna Ab initio Simulation Package (VASP).^114^ The DFT calculations include isolated systems and two kinds of slab systems. The slab systems are 4 layers 3 × 3 Pt(111) slab for the HClO_4_ solution, and 4 layers $\sqrt{3}\times \sqrt{7}$ -R19.1° Pt(111) slab for the H_2_SO_4_ solution. Two bottom layers were fixed, while the top two layers with adsorbates were allowed to relax during the structural optimization. The vacuum layer was set as 30 Å. The cutoff energy was set as 520 eV, which is 1.3 times of the maximum ENMAX of involved elements—oxygen in this study.^115^ The revised Perdew–Burke–Ernzerhof (RPBE) from Hammer et al. was used.^116^ The atomic relaxation convergence standard was set as EDIFFG = −0.02 eV/Å, while the electron step convergence standard was EDIFF = 1 × 10^–6^ eV/cell. The dipole correction along the *z*-direction was considered. The Brillouin zones were sampled using 4 × 4 × 1 and 6 × 4 × 1*k*-points for the (3 × 3) and ( $\sqrt{3}\times \sqrt{7}$ ) supercells, respectively. The Gibbs free energies of all elementary steps are listed in Table S5, which contain the internal energy and the thermal correction including the zero point energy (ZPE) and entropy listed in Tables S1–S4.

### Microkinetic Model for Multistep Reactions

The microkinetic model for serial reaction pathway has been considered in refs 9, 83, 88, 100 and 103 The reaction rate of each elementary step as shown in eqs [Disp-formula eq2-1]–[Disp-formula eq3-5] is expressed as

8 $$v_{i}={\mathrm{k̅}}_{i}^{+}\theta_{i-1}-{\mathrm{k̅}}_{i}^{-}\theta_{i}$$

where ${\mathrm{k̅}}_{i}^{\pm }$ is the forward (+) or backward (−) reaction rate constant considering the concentrations of all non-adsorbed reactants. For example, ${\mathrm{k̅}}_{1}^{+}=k_{1}^{+}{\mathrm{c̃}}_{O_{2}}$ is composed of a reaction rate constant *k*_1_^+^ and a dimensionless (tilde) oxygen concentration referenced to its standard one. *θ*_*i*_ is the coverage of adsorbate generated by the *i* ^th^ step, with *θ*_0_ = *θ*_*N*_ for *N* elementary steps. *θ*_*i*_ conforms to a conservation law, expressed as

9 $$\sum_{i=1}^{N}\theta_{i}=\theta_{\max}$$

where *θ*_max_ is the maximum coverage of active sites for the ORR. For the two adsorbates A and B both generated from the *i* ^th^ step, we used *θ*_*i*_ = *θ*_A+B_ instead of *θ*_A_ *θ*_B_ not only as a result of the consideration to the adjacent condition, but also to avoid the nonlinear terms. The change rate of the adsorbed intermediate coverage is the difference of the reaction rates between two adjacent steps, expressed as

10 $$\frac{d\theta_{i}}{dt}=v_{i}-v_{i+1}$$

which is a set of ordinary differential equations (ODE). At steady state, we have $\frac{d\theta_{i}}{dt}=0$ , eqs 8–10 can be rearranged in a matrix form as *N* = 5 ^83^

11 $$\left[\begin{array}{ccccc} {\mathrm{k̅}}_{1}^{+} & -{\mathrm{k̅}}_{1}^{-}-{\mathrm{k̅}}_{2}^{+} & {\mathrm{k̅}}_{2}^{-} & 0 & 0\\ 0 & {\mathrm{k̅}}_{2}^{+} & -{\mathrm{k̅}}_{2}^{-}-{\mathrm{k̅}}_{3}^{+} & {\mathrm{k̅}}_{3}^{-} & 0\\ 0 & 0 & {\mathrm{k̅}}_{3}^{+} & -{\mathrm{k̅}}_{3}^{-}-{\mathrm{k̅}}_{4}^{+} & {\mathrm{k̅}}_{4}^{-}\\ {\mathrm{k̅}}_{5}^{-} & 0 & 0 & {\mathrm{k̅}}_{4}^{+} & -{\mathrm{k̅}}_{4}^{-}-{\mathrm{k̅}}_{5}^{+}\\ 1 & 1 & 1 & 1 & 1 \end{array}\right]\left[\begin{array}{l} \theta_{0}\\ \theta_{1}\\ \begin{array}{l} \theta_{2}\\ \theta_{3}\\ \theta_{4} \end{array} \end{array}\right]=\left[\begin{array}{l} 0\\ 0\\ \begin{array}{l} 0\\ 0\\ \theta_{\max} \end{array} \end{array}\right]$$

from which the coverages are solved and expressed as

12 $$\theta_{i}=\frac{\theta_{\max}}{\Xi }\left(\frac{1}{{\mathrm{k̅}}_{i+5}^{-}}+\frac{K_{i+5}}{{\mathrm{k̅}}_{i+4}^{-}}+\frac{K_{i+4}K_{i+5}}{{\mathrm{k̅}}_{i+3}^{-}}+\frac{K_{i+3}K_{i+4}K_{i+5}}{{\mathrm{k̅}}_{i+2}^{-}}+\frac{K_{i+2}K_{i+3}K_{i+4}K_{i+5}}{{\mathrm{k̅}}_{i+1}^{-}}\right)$$

with $K_{i}={\mathrm{k̅}}_{i}^{+}/{\mathrm{k̅}}_{i}^{-}$ being the equilibrium constant of the *i* ^th^ elementary step, *K*_*i*_ = *K*_*i*–5_ and ${\mathrm{k̅}}_{i}^{-}={\mathrm{k̅}}_{i-5}^{-}$ as *i* > 5.^83^ Ξ is expressed as

13 $$\Xi =K_{1}K_{2}K_{3}K_{4}K_{5}\left(\frac{\Theta_{1}}{{\mathrm{k̅}}_{1}^{+}}+\frac{\Theta_{2}}{{\mathrm{k̅}}_{2}^{+}}+\frac{\Theta_{3}}{{\mathrm{k̅}}_{3}^{+}}+\frac{\Theta_{4}}{{\mathrm{k̅}}_{4}^{+}}+\frac{\Theta_{5}}{{\mathrm{k̅}}_{5}^{+}}\right)$$

where Θ_*i*_ is a thermodynamic factor, read as

14 $$\Theta_{i}=\frac{1}{K_{i+1}K_{i+2}K_{i+3}K_{i+4}}+\frac{1}{K_{i+2}K_{i+3}K_{i+4}}+\frac{1}{K_{i+3}K_{i+4}}+\frac{1}{K_{i+4}}+1$$

The current density of ORR is expressed as

15 $$j_{ORR}=-e_{0}n_{M}\sum_{i=1}^{5}m_{i}v_{i}$$

where *m*_*i*_ is the number of transferred electrons of the *i* ^th^ step. Defined as the inverse rate of the overall reaction, the overall reaction resistance is expressed as^83^

16 $$R_{ORR}=-\frac{4e_{0}n_{M}\theta_{\max}}{j_{ORR}}\approx \sum_{i=1}^{5}\frac{\Theta_{i}}{{\mathrm{k̅}}_{i}^{+}}$$

where the maximum term in *R*_ORR_ is defined as the RDRT, which incorporates the kinetics and thermodynamic.

The reaction rate constant of the chemical step is described by the transition state theory, read as

17 $$k_{1}^{\pm }=\frac{k_{B}T}{h}\exp\left(-\frac{\Delta G_{a,1}^{\pm }}{k_{B}T}\right)$$

where Δ*G*_a,1_^±^ denote the activation energy of the oxygen adsorption (+) and desorption (−) process with Δ*G*_a,1_^+^ = Δ*G*_a,1_^–^ + Δ*G*_1_^0^, *k*_B_ the Boltzmann constant, *T* the absolute temperature, *h* the Planck constant. The reaction rate of each electron transfer step is described by the Gerischer’s formulation of electron transfer theory^117^

18 $$k_{i}^{+}=\frac{k_{B}T}{h}\int f\left(\varepsilon \right)\rho \left(\varepsilon \right)\frac{1}{\sqrt{4\pi \lambda_{i}k_{B}T}}\exp\left(-\frac{{\left(\lambda_{i}+e_{0}\eta_{i}-\varepsilon \right)}^{2}}{4\lambda_{i}k_{B}T}\right)d\varepsilon$$

19 $$k_{i}^{-}=\frac{k_{B}T}{h}\int \left(1-f\left(\varepsilon \right)\right)\rho \left(\varepsilon \right)\frac{1}{\sqrt{4\pi \lambda_{i}k_{B}T}}\exp\left(-\frac{{\left(\lambda_{i}-e_{0}\eta_{i}+\varepsilon \right)}^{2}}{4\lambda_{i}k_{B}T}\right)d\varepsilon$$

where the Fermi–Dirac distribution *f*(*ε*)= ${\left(1+\exp\left(\left(\varepsilon -E_{F}\right)/k_{B}T\right)\right)}^{-1}$ describes the probability to find an occupied state on the metal surface at energy *ε* referenced to the Fermi level *E*_F_, *ρ*(*ε*) is the density of states (DOS). The exponential term describes the probability of finding the redox species in the solution phase on a certain energy level with a normalizing factor $1/\sqrt{4\pi \lambda_{i}k_{B}T}$ , with *λ*_*i*_ being the reorganization energy. The integral range could from negative infinity to infinity, at least over the needed band of the reaction since the integral is negligible far from the Fermi level. Electrocatalytic effects are built in the Gerischer’s formula via the overpotential of the *i* ^th^ elementary step *η*_*i*_, expressed as

20 $$\eta_{i}=E_{M}-\phi_{OHP}-E_{i}^{eq,0}$$

where *E*_M_ is the electrode potential on the SHE scale, *ϕ*_OHP_ the electric potential at the OHP, *E*_*i*_^eq,0^ the standard equilibrium potential of the *i* ^th^ elementary step, calculated by $-\frac{\Delta G_{i}^{0}}{e_{0}}$ . Δ*G*_*i*_^0^ is the Gibbs free energy under standard conditions at 0 V_SHE_, which vary from catalyst to catalyst. For the intrinsic microkinetic model without the EDL effects, we have *ϕ*_OHP_ = *ϕ* ^b^.

### Mass Transport Model with EDL Effects

The modified Poisson-Nernst–Planck (PNP) equations are expressed as^100^

21 $$\frac{\partial }{\partial x}\left(\epsilon_{s}\frac{\partial \phi }{\partial x}\right)=-e_{0}\sum z_{\alpha }n_{\alpha }$$

22 $$\frac{\partial n_{\alpha }}{\partial t}=-\frac{\partial J_{\alpha }}{\partial x}+R_{\alpha }$$

where the distributions of the electric potential (*ϕ*) and number density of particle (*n*_*α*_) are described from the OHP to the bulk solution while *α* = a, c represents anions and cations. *ϵ*_s_ is an uniform dielectric permittivity, *z*_*α*_ is the charge number of *α*, *J*_*α*_ is the flow flux expressed as

23 $$J_{\alpha }=-D_{\alpha }\frac{n_{s}}{n_{t}}\left(\frac{\partial n_{\alpha }}{\partial x}+\frac{z_{\alpha }e_{0}}{k_{B}T}n_{\alpha }\frac{\partial \phi }{\partial x}+\frac{n_{\alpha }}{n_{s}}\sum_{i\neq s}\frac{\partial n_{i}}{\partial x}\right)$$

The right-hand side represents the diffusion, migration and steric effect. *D*_*α*_ is the diffusion coefficient, *n*_t_ is the total number density for the volume, *n*_s_ is the number density of solvent with *n*_t_=*n*_s_ + ∑*n*_*α*_. The equations come back to the classical PNP equations for dilute solutions, that means *n*_*α*_ ≪ *n*_t_ and *n*_s_≈*n*_t_. We calculate *n*_t_ by 1/*d*_t_^3^ with *d*_t_ being the referenced length of the cubic lattice occupied by a particle. The term *R*_*α*_ describes the dissociation reactions in the electrolyte solution. In the solutions with H_2_SO_4_, there are

24 $$H_{2}SO_{4}⇌H^{+}+HSO_{4}^{-}\left(pK_{a1}=-3\right)$$

25 $$HSO_{4}^{-}⇌H^{+}+SO_{4}^{2-}\left(pK_{a2}=1.99\right)$$

H_2_SO_4_ could be neglected due to the negative p*K*_a1_. *R*_*α*_ is expressed as

26 $$R_{\alpha }=\pm \left(k_{a2}\frac{n_{HSO_{4}^{-}}}{n_{0}}-k_{-a2}\frac{n_{H^{+}}n_{{\mathrm{SO}}_{4}^{2-}}}{n_{0}n_{0}}\right)$$

It is the negative sign for *α* = HSO_4_^–^, and positive sign for *α* = H^+^, SO_4_^2–^. *k*_a2_ and *k*_–a2_ are the rate constants of the forward and backward process, and *n*_0_ is the reference number density set as 1 m^–3^.

The left boundary is set at the OHP, denoted as *x*= 0. The electric potential at the OHP is described by^109^

27 $$\phi \left(0,t\right)=\phi_{OHP}=E_{M}-E_{pzc}-\sigma_{free}\left(\frac{\delta_{IHP}}{\epsilon_{IHP}}+\frac{\delta_{OHP}}{\epsilon_{OHP}}\right)-\frac{\mu_{ad}}{\epsilon_{IHP}}$$

where *E*_pzc_ is the PZC on the SHE scale, *σ*_free_ is the surface free charge density, *δ*_IHP_ is the distance from metal surface to the IHP, *ϵ*_IHP_ is the dielectric permittivity of the space between the metal surface and the IHP, *δ*_OHP_ and *ϵ*_OHP_ are for the space between the IHP and the OHP. *σ*_free_ is defined as the negative total ionic charge density on the solution side,^118,119^ expressed as

28 $$\sigma_{free}\equiv -e_{0}\int_{OHP}^{\infty }\sum_{i}z_{i}n_{i}dx=-{\frac{\partial \phi }{\partial x}}_{{OHP}^{+}}\epsilon_{s}$$

which is also equal to the electric field calculated from the right side of the OHP (OHP^+^) multiplies the dielectric permittivity. $\frac{\mu_{ad}}{\epsilon_{IHP}}$ term is attributed to the surface dipole moment, in which we have

29 $$\mu_{ad}=e_{0}\delta_{IHP}n_{M}\sum_{i}\zeta_{i}\theta_{i}$$

with *e*_0_ being the elementary charge, *n*_M_ the areal number density of metal atoms, *ζ*_*i*_ the number of the electrons taken by each adsorbate, and *θ*_*i*_ the coverage of this adsorbate.

The other left boundary condition is the current-related flux at the OHP, read as

30 $$J_{\alpha }\left(0,t\right)=-n_{M}v_{\alpha }$$

where *v*_*α*_ is the sum of the reaction rates related to the particle α calculated by the microkinetic model. The negative sign means the assumption of *α*.

The right boundary is in the bulk solution, denoted as *x*=*x*_r_. There are the following natural boundary conditions

31 $$\phi \left(x_{r},t\right)=0$$

32 $$n_{\alpha }\left(x_{r},t\right)=n_{\alpha }^{b}$$

The electric potential in the solution bulk is taken as the reference. *n*_*α*_^b^ is the number density of particle *α* in the solution bulk. *x*_r_ is the thickness of the diffusion layer, which is calculated as 9.76 μm at 2500 rpm as introduced in Table S12.

### Hierarchical Theoretical Model

The hierarchical theoretical model consists of three interconnected parts. The DFT calculation provides the reaction mechanism for the microkinetic model. The microkinetic model treats the interplay between multiple elementary reactions without designating a rate-determining step.^83,120^ The obtained current density is then used in the boundary condition for the third component describing mass transport in the electrolyte solution. The mass transport model is formulated using modified PNP equations considering diffusion, migration, steric effects and dissociation reactions in solution.^9,100^ The electrolyte solution region solved in this model has a thickness of tens of micrometers, extending from the reaction plane in the EDL to the outer diffusion layer. In turn, the local electric potential and concentrations at the reaction plane are used in the kinetic rate expressions in the microkinetic model. The present hierarchical model is self-consistently calculated under nonequilibrium conditions. The implementation of the numerical simulation is via COMSOL software, with the parameters listed in Supporting Information.

## Supplementary Material



## Data Availability

All data are available from the author upon request.

## Notes

Experimental results on the RHE scale; Comparison of the experimental results; DFT calculation details; Comparisons of the Gibbs free energies; Intrinsic kinetic model parameters and results; Mass transport model parameters and results; Further DFT calculation details (PDF)
