# Supplementary material for: Proton-Relaying Adsorbates Induce Non-Nernstian Behavior in Oxygen Reduction
Source: ACS Catal. 2025 Aug 1;15(16):14191–206. doi: 10.1021/acscatal.5c01767 (PMC12362434; doi:10.1021/acscatal.5c01767)
Supplement: Supplementary file 1 [file cs5c01767_si_001.pdf]

Supporting information for

Proton-Relaying Adsorbates Induce Non-Nernstian Behavior in Oxygen Reduction

Lulu Zhang,[1,2,3] Dongchen Zhao,[1] Weiqiang Tang,[2] Yanxia Chen,[1,*] Jun Huang,[2,3,*]

1 Hefei National Research Center for Physical Sciences at Microscale, Department of Chemical Physics, University of Science and Technology of China, Hefei, 230026, China

2 Institute of Energy Technologies, IET-3: Theory and Computation of Energy Materials, Forschungszentrum Jülich GmbH, 52425 Jülich, Germany

3 Theory of Electrocatalytic Interfaces, Faculty of Georesources and Materials Engineering, RWTH Aachen University, 52062 Aachen, Germany

** Corresponding authors, email: ju.huang@fz-juelich.de, yachen@ustc.edu.cn*

1. Electrochemical experiments

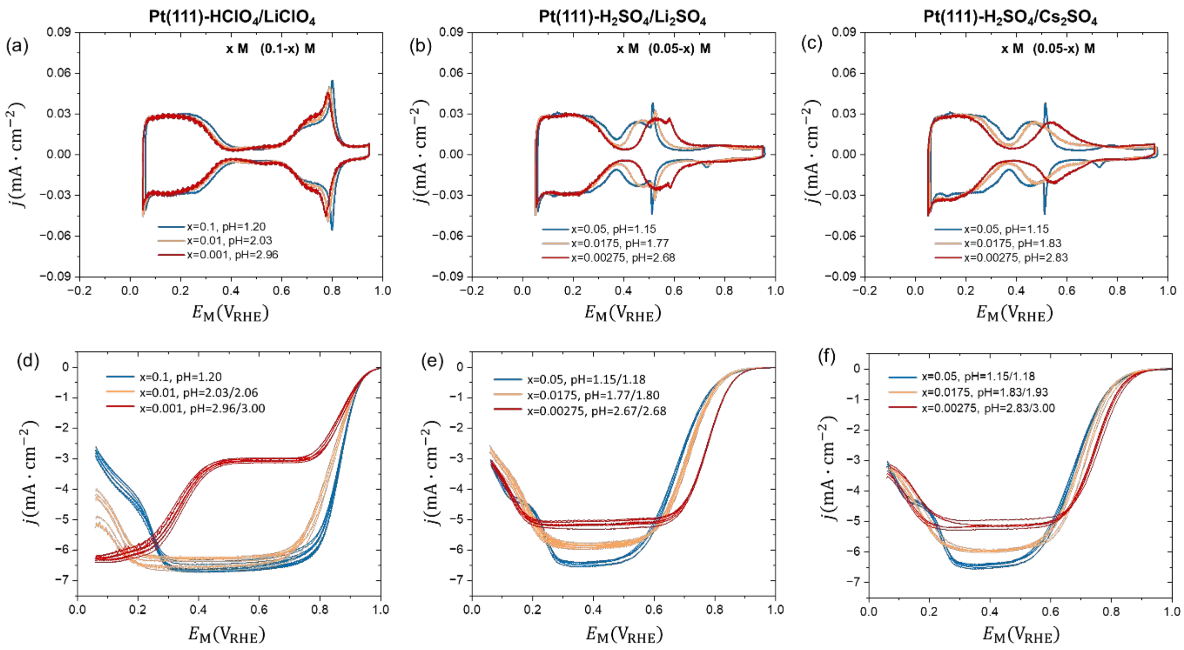

Fig.S1 The same data in Fig.1 are now plotted at the reversible hydrogen electrode (RHE) scale.

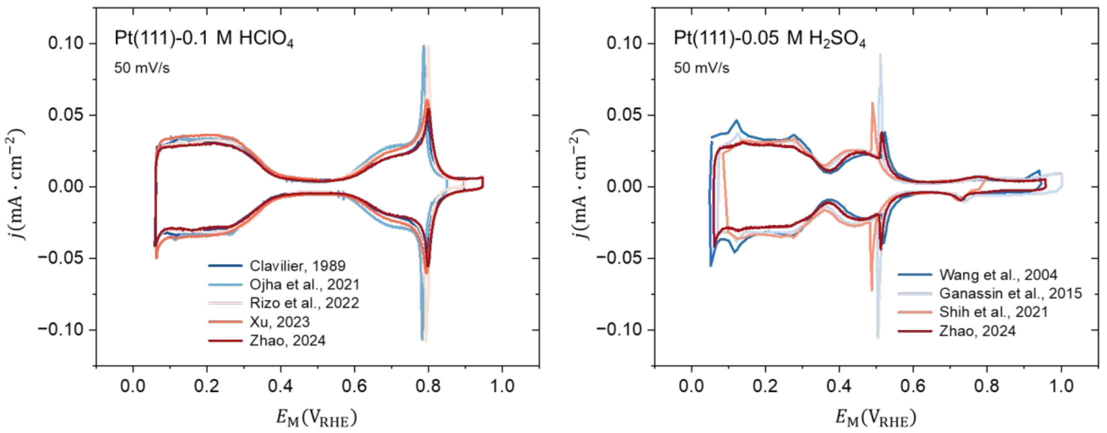

Fig.S2 Comparisons between the cyclic voltammograms of (a) Pt(111)-0.1 M $HClO_4$ interface including Clavilier,[1] Ojha et al.,[2] Rizo et al.,[3] Xu et al.,[4] and this work, and (b) that of Pt(111)-0.05 M H_2SO_4 interface including Wang et al.,[5] Ganassin et al.,[6] Shih et al.,[7] and this work.

2. DFT calculation

2.1 Energy of isolated systems

Table S1. Energies of the isolated systems

Isolated system	Internal energy U/eV	Thermal corrections, eV	Condition	Gibbs free energy G^0/eV
H_2	-7.06	-0.14	1 atm	-7.20
H_2O	-14.75	0.00	0.035 atm	-14.75
O_2	$G^0(O_2) = 4.92 + 2G^0(H_2O) - 2G^0(H_2)$		1 atm	-10.18

Note: 0.035 atm is the equilibrium pressure for gas phase water in contact with liquid water at 298 K.

Table S2. The thermal corrections of the isolated systems from several sources

	Source	Corrections/eV	H_2	H_2O
1	Used in Ref.[8]. The ZPE and the entropy term TS are from a textbook.[9] The thermal correction is obtained by ZPE-TS.	ZPE	0.27	0.56
		TS	0.41	0.67
		ZPE-TS	**-0.14**	**-0.11**
2	Used in Ref.[10]. The ZPE values are calculated with the Phonon-5.0.2 module in espresso-5.0, and the entropy values are from a handbook.[11] The thermal correction is obtained by ZPE-TS.	ZPE	0.27	0.59
		TS	0.41	0.67
		ZPE-TS	**-0.14**	**-0.08**
3	Used in Ref.[12], which is from the vibration analysis. The thermal correction is calculated by H-TS, where H is the enthalpy.	H	0.359	0.668
		TS	0.403	0.584
		H-TS	**-0.044**	**0.084**
4	Calculated by Vaspkit by us.[13] The thermal correction contains the ZPE, TS and the energy change due to temperature and pressure changes, expressed by ZPE+delta_U(0->T)+PV-TS.	ZPE	0.270	0.568
		TS	0.402	0.670
		delta_U(0->T)	0.064	0.077
		PV	0.026	0.026
		ZPE+delta_U(0->T)+ PV-TS	**-0.042**	**0.001**
5	This work		**-0.14**	**0.00**

2.2 Energy of the slab systems

Table S3. Internal energies of the slab systems

Electrode	Adsorption	Internal energy U/eV
4 layers (3x3) Pt(111) slab	$6H_2O*$	-430.34
	O_2*+6H_2O*	-440.66
	O_2**+5H_2O*	-426.26
	O_2**+4H_2O*	-410.57
	$OOH*+5H_2O*$	-430.42
	$OOH**+4H_2O*$	-414.98
	$O*_h+6H_2O*$	-436.26
	$OH*+5H_2O*$	-425.95
4 layers ($\sqrt{3}\times\sqrt{7}$)-R19.1° Pt(111) slab	SO_4*	-220.61
	H_2O*+SO_4*	-236.53
	$2H_2O*+SO_4*$	-252.32
	O_2**+SO_4*	-231.74
	$O_2*+H_2O*+SO_4*$	-246.64
	$OOH**+SO_4*$	-236.27
	$OOH*+H_2O*+SO_4*$	-251.45
	$2OH*+SO_4*$	-242.09
	$O*+H_2O*+SO_4*$	-241.65
	$OH*+O*+SO_4*$	-236.24
	$OH*+H_2O*+SO_4*$	-247.27

Table S4. The thermal corrections of the slab systems from several sources

	Source	Corrections/eV	H_2O*	O*	OH*	O_2*	OOH*
1	Used in Ref.[8]. The ZPE is taken from DFT calculations on Cu(111). The thermal correction is assumed as ZPE.	ZPE		**0.07**	**0.30**		
2	Used in Ref.[10]. The ZPE values are calculated with the Phonon-5.0.2 module in espresso-5.0. The thermal correction is assumed as ZPE.	ZPE		**0.08**		**0.15**	**0.47**
3	Used in Ref.[12], which is from the vibration analysis. The thermal correction is calculated by H-TS, where H is the enthalpy.	H	0.723	0.098	0.400		0.527
		TS	0.149	0.041	0.130		0.144
		H-TS	**0.574**	**0.057**	**0.270**		**0.383**
4	Used in Ref.[14]. The corrections include the ZPE and TS, which are from DFT calculation of the vibrational frequencies.			**0.05**	**0.35**		**0.4**
5	This work for Pt(111)-$HClO_4$ interface		**0.49**	**0.05**	**0.35**	**0.10**	**0.40**
	This work for Pt(111)-H_2SO_4 interface		**0.56**	**0.05**	**0.35**	**0.10**	**0.40**

2.3 Gibbs free energy

The Gibbs free energy of each elementary step under standard conditions at 0 V_{SHE} is calculated by $\Delta G_i^0 = \sum G^0(\text{product}) - \sum G^0(\text{reactant})$. The energy is for the system including the adsorbed specie, solvent, oxygen, proton and electron, if any. Taking the reaction $\mathrm{O_2} + 6\mathrm{H_2O}* \rightleftharpoons (\mathrm{O_2} + 6\mathrm{H_2O})*$ as an example, we have $\Delta G_1^0 = G^0((\mathrm{O_2} + 6\mathrm{H_2O})*) - G^0(6\mathrm{H_2O}*) - G^0(\mathrm{O_2})$, where the Gibbs free energy is obtained by the internal energy and the thermal corrections as listed in Table S1 to S4.

Table S5. The Gibbs free energy of each elementary step under standard conditions at 0 V_{SHE}

	Elementary step (simple expression)	$\Delta G_i^0/\mathrm{eV}$
Pt(111)-$HClO_4$	$O_2 + * \rightleftharpoons O_2 *$	**-0.04**
	$O_2 + H_2O * \rightleftharpoons O_2 ** + H_2O$	-0.88
	$O_2 + 2H_2O * \rightleftharpoons O_2 ** + 2H_2O$	-0.43
	$O_2 * + H_2O * + H^+ + e^- \rightleftharpoons OOH * + H_2O + *$	**-1.10**
	$O_2 ** + H^+ + e^- \rightleftharpoons OOH * + *$	-0.26
	$O_2 ** + H_2O * + H^+ + e^- \rightleftharpoons OOH ** + H_2O + *$	-0.06
	$OOH * + H^+ + e^- \rightleftharpoons O*_h + H_2O *$	**-2.11**
	$O*_h + H_2O * + H^+ + e^- \rightleftharpoons OH * + H_2O$	**-1.02**
	$OH * + H^+ + e^- \rightleftharpoons H_2O *$	**-0.65**
Pt(111)-H_2SO_4	$H_2O + * \rightleftharpoons H_2O *$	-0.61
	$H_2O + H_2O * + * \rightleftharpoons 2H_2O *$	-0.49
	$O_2 + 2H_2O * \rightleftharpoons O_2 ** + 2H_2O$	**0.25**
	$O_2 + 2H_2O * \rightleftharpoons O_2 * + H_2O * + H_2O$	0.65
	$O_2 ** + H^+ + e^- \rightleftharpoons OOH **$	**-0.63**
	$O_2 ** + H^+ + e^- + H_2O \rightleftharpoons OOH * + H_2O *$	-0.5
	$OOH ** + H^+ + e^- \rightleftharpoons 2OH *$	**-1.92**
	$OOH ** + H^+ + e^- \rightleftharpoons O * + H_2O *$	-1.57
	$OOH ** \rightleftharpoons O * + OH *$	0.03
	$2OH * + H^+ + e^- \rightleftharpoons OH * + H_2O *$	**-1.38**
	$OH * + H_2O * + H^+ + e^- \rightleftharpoons 2H_2O *$	**-1.24**

*The elementary steps with bold values are the chosen steps.

Table S6. Gibbs free energy (eV) profile of ORR at Pt(111)-$HClO_4$ interface under standard conditions at 0 V_{SHE}

Source	O_2* O_2**	OOH*	$O*_h$	OH*	Exchange-correlation functional	Pt size	Solvent	Adsorbate coverage
Nørskov et al., 2004 [8]	5.59	4.66	2.41	1.76	RPBE	3×(3×2)	Bilayer of explicit waters are on the top of adsorbates.	1/2 OX*
Panchenko et al., 2004 [15]	4.46/4.4 (O_2**_h) 4.29 (O_2**)			0.52	PW91	4×(2×2)	On bare surface	1/4
Karlberg et al., 2007 [16]		4.13	1.6	0.81	RPBE	3×(3×2)	One water for per OH* and OOH*	1/2 OH*/OOH* and 1/2 H_2O*
Tripkovic´ et al., 2010 [17]	5.01 (O_2**)	3.95		0.79	RPBE	3 layers	Bilayer of explicit waters	2/3 (OH*+H_2O*) Reference to (OH*+5H_2O*)
Hansen et al., 2014 [18]	4.99 (O_2*)	3.91	1.7	0.75	RPBE	3×(3×2$\sqrt{3}$)	One layer of explicit waters	1/3 OH* to 1/3 O*
Eslamibidgoli et al., 2015 [19]		4.09	1.37	0.84	PBE	4×(3×3)	One layer of explicit waters with the H-up structure	2/3 (OX*+H_2O*)
		4.12	1.34	0.59			… H-down structure	
Liu et al., 2016 [20]	4.64	4.42	1.44	1.26	PW91	4×(3×3)	On bare surface	2/3 (OX*+H_2O*)
	4.37 (O_2**)	3.87	1.50	0.67			One bilayer of explicit water	
	4.52	3.71	1.40	0.60			Two bilayers of explicit water	
	4.50	3.62	1.40	0.68			Three bilayers of explicit water	
Haile et al., 2020 [21]		4.08	1.52	0.84	RPBE	4×(2×2)	The implicit solvation model was implemented in VASPsol, with the dielectric constant as 78.54.	1/4
Our results	4.88 (O_2*) 4.04 (O_2**)	3.78	1.67	0.65	RPBE	4×(3×3)	One layer of explicit waters	2/3 (OX*+H_2O*)

2.4 Supplement to the reaction pathways

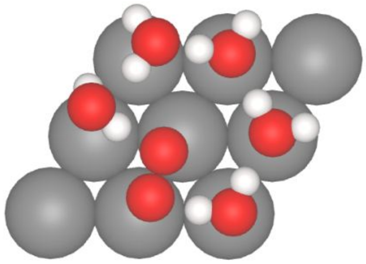

Fig.S3. The structure of $(O_2 ** + 5H_2O *)$ at 4 layers (3×3) Pt(111). Only Pt atoms in the first layer are shown. Pt is grey, O red and H white, which are set up via the VESTA software.[22]

For the 4 layers (3x3) Pt(111) slab, we also observed that the O* on the top site prefers to combine with the adjacent OH* or O* to form a O-O bond spontaneously. Therefore, the oxygen decomposition step, $O_2 * \rightarrow 2O *$, and the OOH* decomposition step, $OOH * \rightarrow O * + OH *$ are neglected. When the O* on the top site is close to one water adsorbate, two OH* adsorbates are produced, that is, $O * + H_2O * \rightleftharpoons 2OH *$. Since $O *$ prefers to stay on the hollow site with a lower energy, this step is not considered in this work.

3. Intrinsic kinetic model

Table S7. General constants

Symbol	Value	Physical significance
k_B	1.38×10^{-23} J K^{-1}	Boltzmann constant
T	298.15 K	Absolute temperature
e_0	1.60×10^{-19} C	Elementary charge
h	6.63×10^{-34} J s	Planck constant
N_A	6.02×10^{23} mol^{-1}	Avogadro constant
ϵ_0	8.85×10^{-12} F m^{-1}	Vacuum permittivity

Table S8. Dimensionless concentrations of ORR reactants in the bulk solution

Symbol	Value	Physical significance
$\tilde{c}_{O_2}^{b}$	0.032	The oxygen concentration in the bulk solution referenced to its standard concentration at 1 atm, expressed as $\tilde{c}_{O_2}^{b} = \frac{c_{O_2}^{b}}{c_{O_2}^{gas}}$ with $c_{O_2}^{gas}$=0.0403 M. According to the Henry law, the value of $\tilde{c}_{O_2}^{b}$ is the Henry constant at 298 K as 0.032.
$\tilde{c}_{H^+}^{b}$	0.1, 0.01, 0.001 (*HClO₄*)	The proton concentration in the bulk solution $c_{H^+}^{b}$ referenced to its standard concentration c_0 as 1 M.
	0.057, 0.01, 0.001 (*H₂SO₄*)	Based on the equilibrium relationship of $HSO_4^- \rightleftharpoons H^+ + SO_4^{2-}$ with $pK_{a2} = 1.99$, we have $\frac{c_{H^+}^{b} c_{SO_4^{2-}}^{b}}{c_{HSO_4^-}^{b} c_0} = K_{a2}$. Based on the element conservation of S and H, we have $c_{SO_4^{2-}}^{b} + c_{HSO_4^-}^{b} = c_{H_2SO_4}^{b} + c_{Li_2SO_4}^{b}$ and $c_{H^+}^{b} + c_{HSO_4^-}^{b} = 2\, c_{H_2SO_4}^{b}$. $c_{H^+}^{b}$ could be calculated, and $\tilde{c}_{H^+}^{b} = c_{H^+}^{b}/c_0$.
$\tilde{c}_{H_2O}$	1	The water concentration in the bulk solution $c_{H_2O}^{b}$ referenced to its standard concentration as 55.6 M.

Table S9. Parameters of the intrinsic kinetic model

Symbol	Value	Physical significance	Ref
n_{M}	$1.5 \times 10^{19}\ \mathrm{m}^{-2}$	Areal number density of Pt atoms, calculated by $4/(\sqrt{3}\,a_{\mathrm{Pt}}{}^{2})$ with the lattice constant as $a_{\mathrm{Pt}} = 3.92\mathrm{Å}$	[23]
ε	[-6,6] eV	The considered metal band region. The electrons with even lower energy and the orbitals with higher energy can be disregarded.	[24]
E_{F}	0 eV	Fermi level of the metal	[24]
k_{M}	$0.05\ \mathrm{V\ s^{-1}}$	Scanning rate. The ORR performance is evaluated using the linear scanning method. The electrode electric potential changes with time according to $E_{\mathrm{M}} = E_{\mathrm{M,begin}} + \mathrm{sign}\left(E_{\mathrm{M,end}} - E_{\mathrm{M,begin}}\right) \times k_{\mathrm{M}} t$, where $E_{\mathrm{M,begin}}$ is the starting potential, $E_{\mathrm{M,end}}$ is the ending potential, and the sign function determines the direction of the potential scanning (positive or negative).	Exp
λ_0	1.75 eV (*$HClO_4$*)<br>0.92 eV (*H_2SO_4*)	Reorganization energy of all electron transfer steps at pH 0	Fitted
$\Delta G^{+}_{a,1}$	0.15 eV (*$HClO_4$*)<br>0.4 eV, 0.6 eV (*H_2SO_4*)	Activation energy of the oxygen adsorption process	For analysis
$\theta_{\max}$	2/3 (*$HClO_4$*)<br>1/5 (*H_2SO_4*)	Maximum active sites for ORR. 1/5 instead of 2/5 is due to the $\theta_{\mathrm{A+B}}$ instead of $\theta_{\mathrm{A}}\theta_{\mathrm{B}}$ is used.	DFT

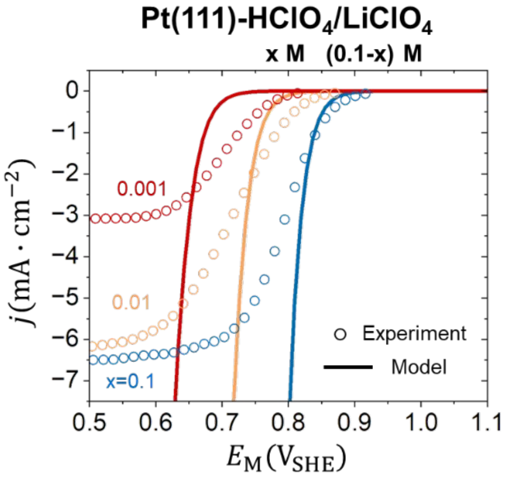

Fig.S4 The ORR polarization curves obtained by experiment (circles) and intrinsic kinetic model (curves) where the reorganization free energy is pH independent at Pt(111) in x M $HClO_4$+(0.1-x) M $LiClO_4$ (x=0.1, 0.01, 0.001) solutions.

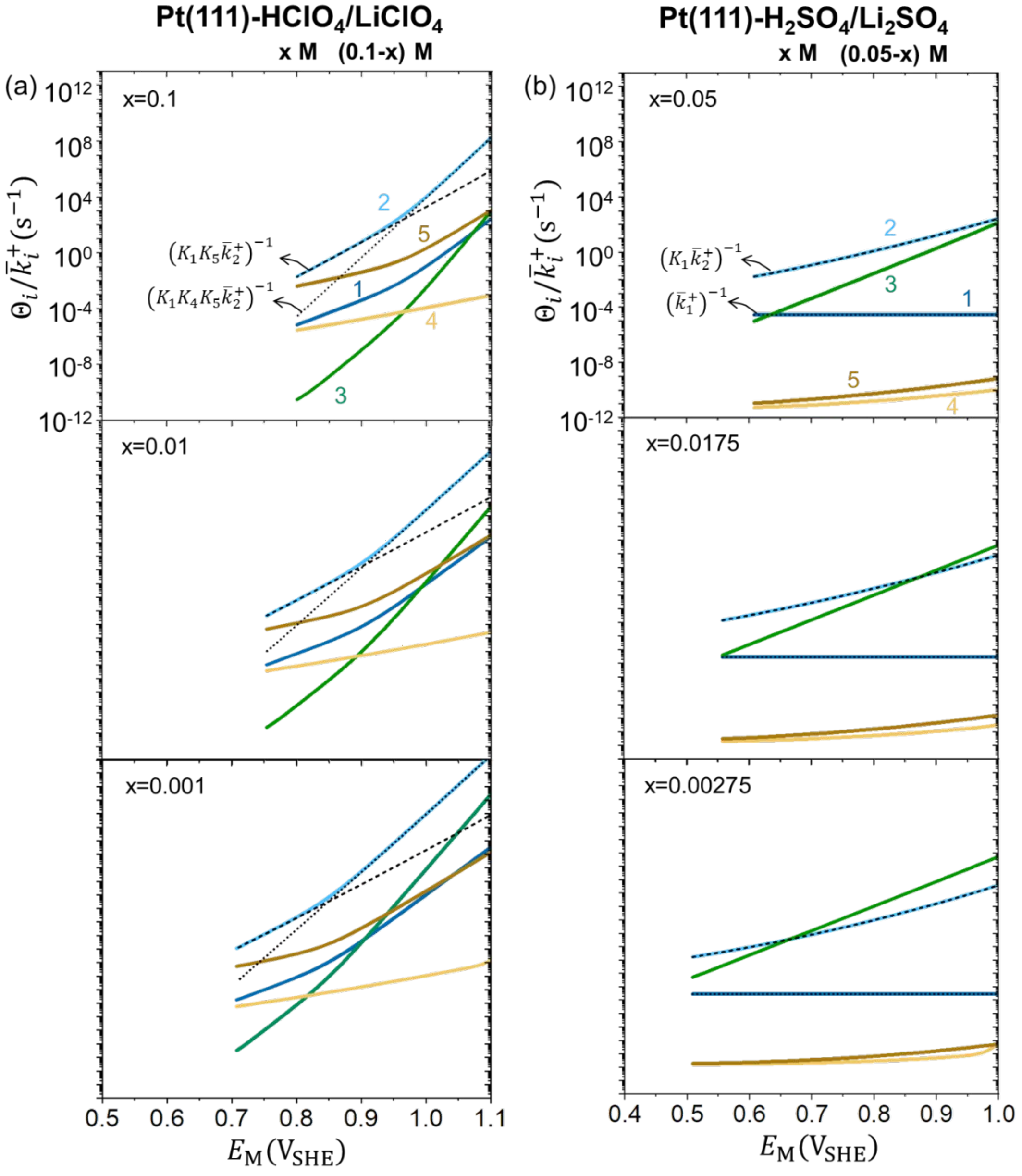

Fig.S5 The resistive terms of $\frac{\Theta_i}{\bar{k}_i^+}$ obtained by the intrinsic kinetic model in (a) x M $HClO_4$+(0.1-x) M $LiClO_4$ (x=0.1, 0.01, 0.001) and (b) x M H_2SO_4+(0.05-x) M Li_2SO_4 (x=0.05, 0.0175, 0.00275) solutions.

4. Mass transport model with EDL effects

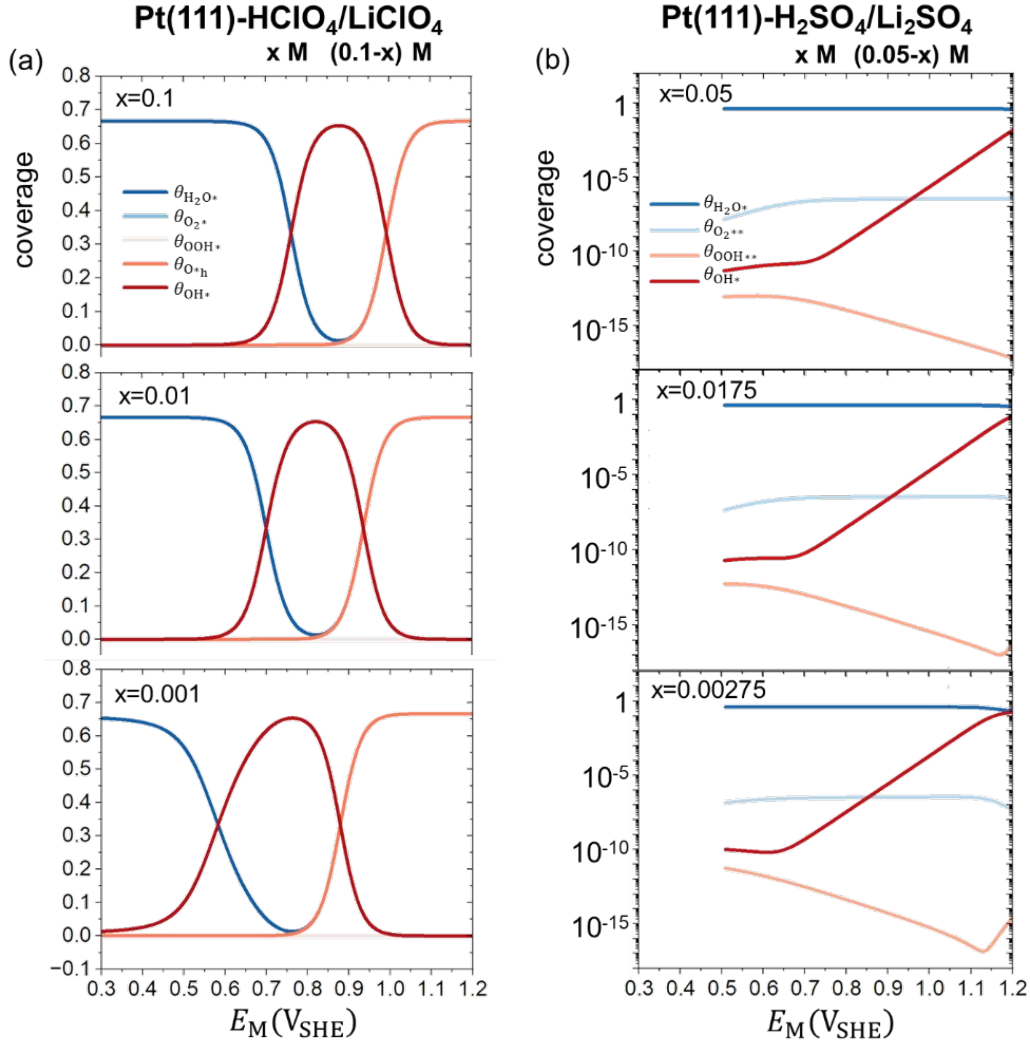

Fig.S6 Potential dependent coverages of the reaction intermediates of ORR at Pt(111) in (a) x M $HClO_4$+(0.1-x) M $LiClO_4$ (x=0.1, 0.01, 0.001) and (b) x M H_2SO_4+(0.05-x) M Li_2SO_4 (x=0.05, 0.0175, 0.00275) solutions, obtained by the hierarchical theoretical model.

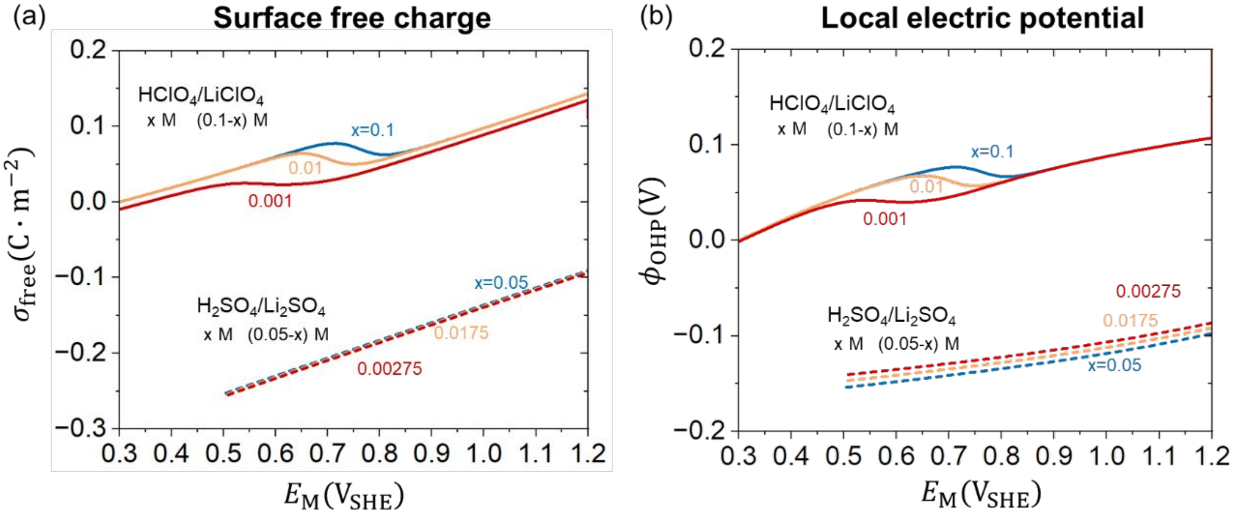

Fig.S7 The potential-dependent (a) surface free charge density and (b) local electric potential at Pt(111) in x M $HClO_4$+(0.1-x) M $LiClO_4$ (x=0.1, 0.01, 0.001) solutions (curves) and x M H_2SO_4+(0.05-x) M Li_2SO_4 (x=0.05, 0.0175, 0.00275) solutions (dashed lines), obtained by the hierarchical theoretical model.

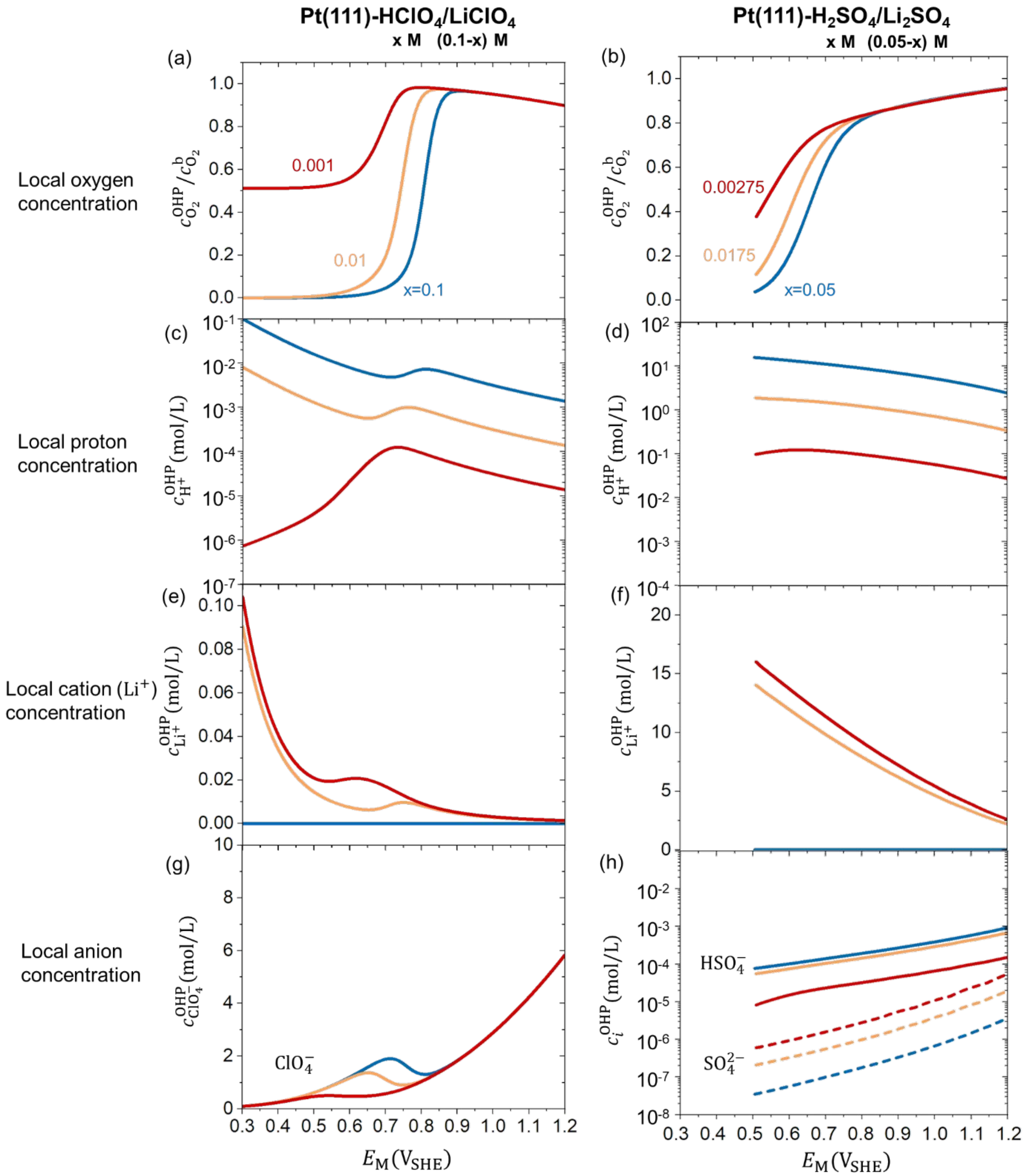

Fig.S8 The electrode potential dependent local concentration of (a,b) oxygen, (c,d) proton, (e,f) cation and (g,h) anion(s) in (left) x M $HClO_4$+(0.1-x) M $LiClO_4$ (x=0.1, 0.01, 0.001) and (right) x M H_2SO_4+(0.05-x) M Li_2SO_4 (x=0.05, 0.0175, 0.00275) solutions, obtained by the hierarchical theoretical model.

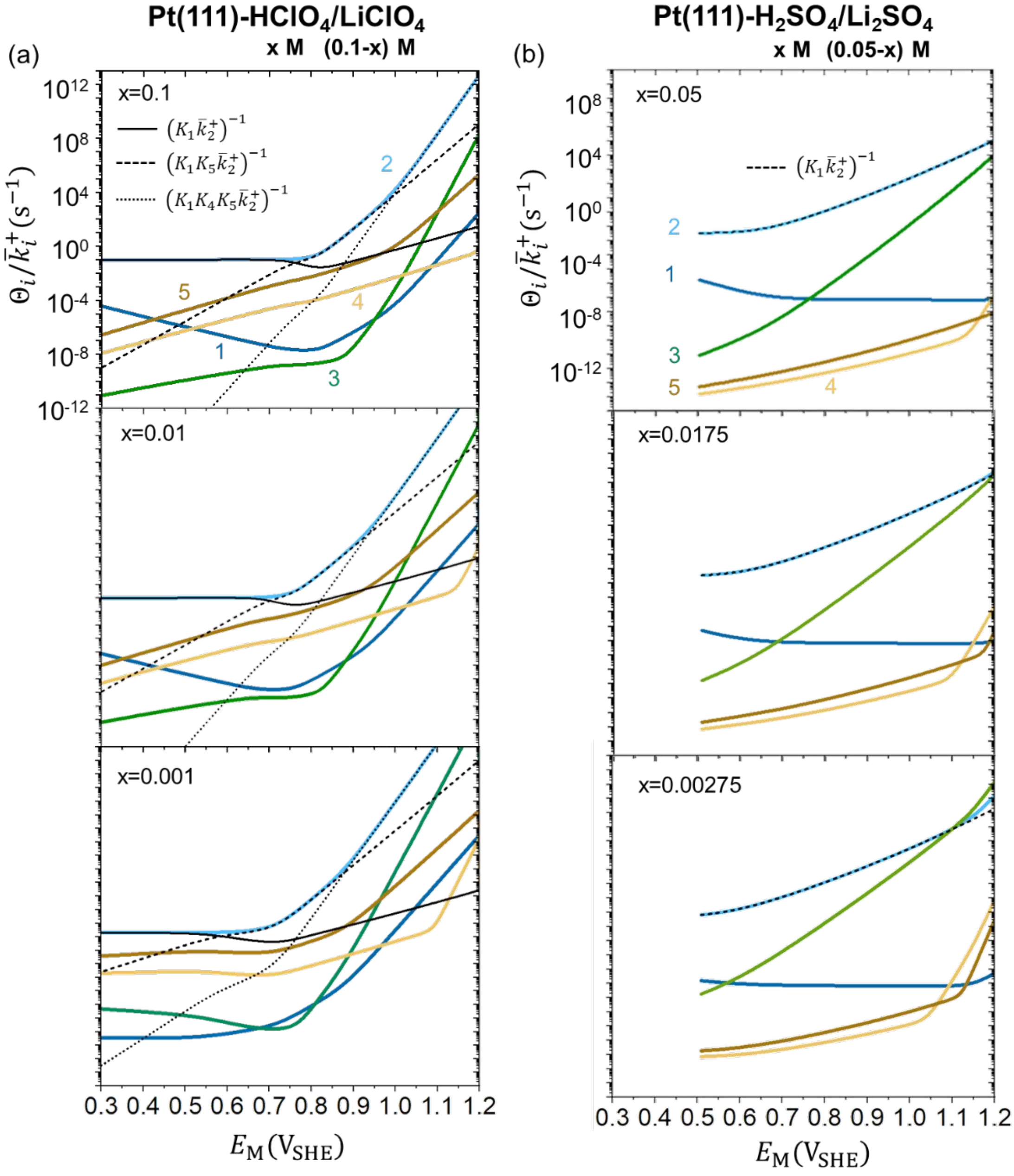

Fig.S9 The resistive terms of $\frac{\Theta_i}{\bar{k}_i^+}$ in (a) x M $HClO_4$+(0.1-x) M $LiClO_4$ (x=0.1, 0.01, 0.001) and (b) x M H_2SO_4+(0.05-x) M Li_2SO_4 (x=0.05, 0.0175, 0.00275) solutions, obtained by the hierarchical theoretical model.

Table S10. Parameters of the electrical double layer

Symbol	Value	Physical significance	Ref
ϵ_{IHP}	$6\epsilon_0$	Dielectric permittivity of the space between the metal and the IHP	[25]
ϵ_{OHP}	$30\epsilon_0$	Dielectric permittivity of the space between the IHP and the OHP	[25]
ϵ_{s}	$78.5\epsilon_0$	Dielectric permittivity of the water solvent medium	[26]
δ_{IHP}	0.15 nm	Distance from the metal surface to the IHP	[25]
δ_{OHP}	0.3 nm	Distance from the IHP to the OHP	[25]
ϕ_{pzc}	0.3 $\mathrm{V_{SHE}}$	Potential of zero free charge	[27]
$\zeta_{\mathrm{OH*}}, \zeta_{\mathrm{O*}}, \zeta_{\mathrm{OOH*}}$	0.05	Average electron number of OH*,O* and OOH*	
$\zeta_{\mathrm{SO_4*}}$	1	Average electron number of adsorbed sulfate anion	[28]

Table S11. Dimensionless concentrations of ions in the bulk solution

Symbol	Value	How to obtain
in x M $HClO_4$+(0.1-x) M $LiClO_4$ solutions as x=0.1, 0.01, 0.001		
$\tilde{c}^{\mathrm{b}}_{\mathrm{Li^+}}$	0, 0.09, 0.099	$\tilde{c}^{\mathrm{b}}_i = c^{\mathrm{b}}_i / c_0$ with $c_0 = 1$ M.
$\tilde{c}^{\mathrm{b}}_{\mathrm{ClO_4^-}}$	0.1	
in x M H_2SO_4+(0.05-x) M Li_2SO_4 solutions as x=0.05, 0.0175, 0.00275		
$\tilde{c}^{\mathrm{b}}_{\mathrm{HSO_4^-}}$	0.043, 0.025, 0.0045	Based on the equilibrium relationship of $\mathrm{HSO_4^-} \rightleftharpoons \mathrm{H^+} + \mathrm{SO_4^{2-}}$ with $\mathrm{p}K_{\mathrm{a2}} = 1.99$, we have $\frac{c^{\mathrm{b}}_{\mathrm{H^+}} c^{\mathrm{b}}_{\mathrm{SO_4^{2-}}}}{c^{\mathrm{b}}_{\mathrm{HSO_4^-}} c_0} = K_{\mathrm{a2}}$. Based on the element conservation of S and H, we have $c^{\mathrm{b}}_{\mathrm{SO_4^{2-}}} + c^{\mathrm{b}}_{\mathrm{HSO_4^-}} = c^{\mathrm{b}}_{\mathrm{H_2SO_4}} + c^{\mathrm{b}}_{\mathrm{Li_2SO_4}}$ and $c^{\mathrm{b}}_{\mathrm{H^+}} + c^{\mathrm{b}}_{\mathrm{HSO_4^-}} = 2c^{\mathrm{b}}_{\mathrm{H_2SO_4}}$. c^{b}_i could be calculated, and $\tilde{c}^{\mathrm{b}}_i = c^{\mathrm{b}}_i / c_0$ with $c_0 = 1$ M.
$\tilde{c}^{\mathrm{b}}_{\mathrm{SO_4^{2-}}}$	0.007, 0.025, 0.0455	
$\tilde{c}^{\mathrm{b}}_{\mathrm{Li^+}}$	0, 0.065, 0.0945	

Table S12. Parameters of the mass transport model

**Symbol**	**Value**	**Physical significance**	**Ref**
x_r	9.76 μm	Thickness of the diffusion layer, calculated by, $x_\mathrm{r} = 1.61D^{1/3}$ $(\eta/\rho)^{1/6}\omega^{-1/2}$, where $D = 1 \times 10^{-9}\ \mathrm{m^2 s^{-1}}$ is the typical diffusion coefficient, η the viscosity of water, ρ the density of water, and ω the rotate speed.	29
ω	$2500\ \mathrm{rpm}$	Rotate speed	Exp
η	$8.91 \times 10^{-4}\ \mathrm{Pa\ s}$	Viscosity of water	9
ρ	$997\ \mathrm{kg\ m^{-3}}$	Density of water	9
$D_{\mathrm{O_2}}$	$1.25 \times 10^{-9}\ \mathrm{m^2\ s^{-1}}$	Oxygen diffusion coefficient, fitted	30
$D_{\mathrm{H^+}}$	$3.1 \times 10^{-9}\ \mathrm{m^2\ s^{-1}}$	Proton diffusion coefficient, fitted	30
k_{a2}	$10^6\ \mathrm{m^{-3}s^{-1}}$	Forward reaction rate of the $\mathrm{HSO_4^-}$ dissociation reaction. We take $k_{\mathrm{a2}} = 10^6\ \mathrm{m^{-3}s^{-1}}$ and $k_{-\mathrm{a2}} = \frac{k_{\mathrm{a2}}}{K_{\mathrm{a2}}}$. This value is close to a similar dissociation reaction.	31
d_t	0.31 nm	Length of the reference cell, calculated by $(55.6 \times 10^3 N_\mathrm{A})^{-1/3}$	

Table S13. Energy parameters

**Symbol**	**Value**	**Physical significance**
in x M HClO$_4$+(0.1-x) M LiClO$_4$ solutions as x=0.1, 0.01, 0.001		
λ_0	2 eV	Reorganization energy, fitted
$\Delta G^0_{\mathrm{O_2*}}$	5.05 eV	Standard Gibbs free energy of adsorption systems, fitted according to the Tafel slope
$\Delta G^0_{\mathrm{OOH*}}$	3.78 eV	
$\Delta G^0_{\mathrm{O*_h}}$	1.87 eV	
$\Delta G^0_{\mathrm{OH*}}$	0.82 eV	
$\Delta G^+_{a,1}$	0.15 eV	Activation energy of oxygen adsorption process, taken as a typical value
in x M H$_2$SO$_4$+(0.05-x) M Li$_2$SO$_4$ solutions as x=0.05, 0.0175, 0.00275		
λ_0	1.4 eV	Reorganization energy, fitted
$\Delta G^+_{a,1}$	0.3 eV	Activation energy of oxygen adsorption process

5. Proton relay theory

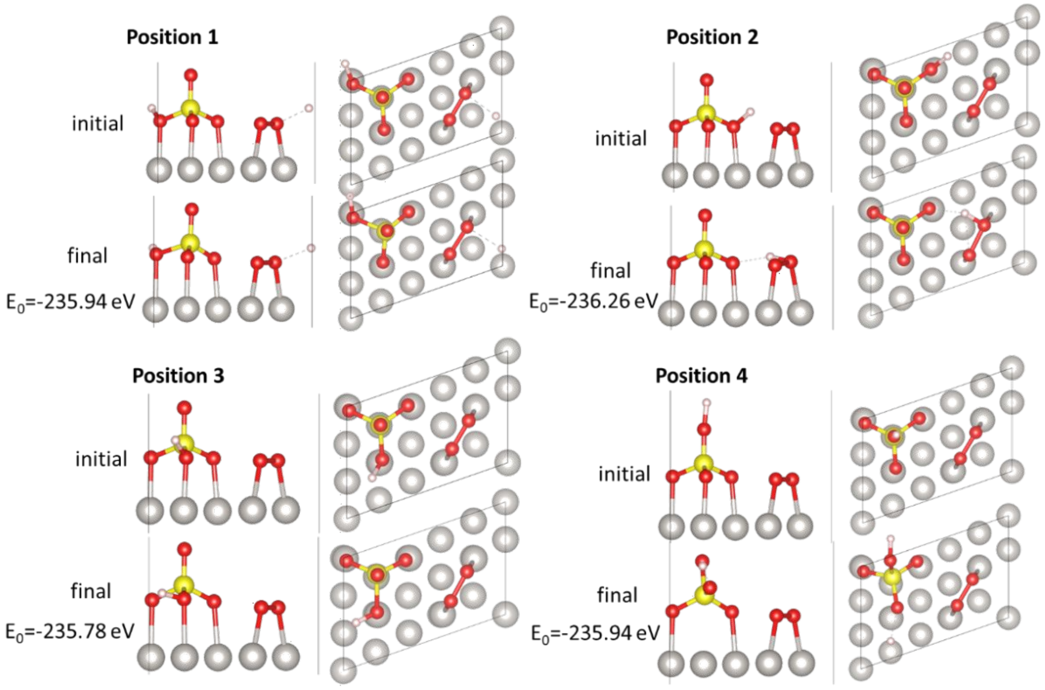

Fig.S10 Four positions for a proton to connect with an adsorbed sulfate to form one bisulfate adsorbate. Pt is grey, O red, H white and S yellow.

The proton at the first position has a stronger interaction with the adsorbed sulfate rather than adsorbed oxygen. The proton at the second position prefers to connect with adsorbed oxygen to form OOH adsorbate. The proton at the third position prefers to stay with adsorbed sulfate. The proton at the last position changes the structure of adsorbed sulfate.

Table S14. Parameters for the proton relay theory

Symbol	Value	Physical significance
in x M H_2SO_4+(0.05-x) M Li_2SO_4 solutions as x=0.05, 0.0175, 0.00275		
α	0.5	Reaction order of proton
λ_0	1.35 eV	Reorganization energy, fitted
in x M H_2SO_4+(0.05-x) M Cs_2SO_4 solutions as x=0.05, 0.0175, 0.00275		
α	0.75	Reaction order of proton
λ_0	1.375 eV	Reorganization energy, fitted

References

(1) Clavilier, J. The Role of Anion on the Electrochemical Behaviour of a {111} Platinum Surface; an Unusual Splitting of the Voltammogram in the Hydrogen Region. *J. Electroanal. Chem. Interf. Electrochem.* **1980**, *107* (1), 211-216.

(2) Ojha, K.; Doblhoff-Dier, K.; Koper, M. T. M. Double-Layer Structure of the Pt(111)–Aqueous Electrolyte Interface. *P. Natl. Acad. Sci.* **2022**, *119* (3), e2116016119.

(3) Rizo, R.; Fernández-Vidal, J.; Hardwick, L. J.; Attard, G. A.; Vidal-Iglesias, F. J.; Climent, V.; Herrero, E.; Feliu, J. M. Investigating the Presence of Adsorbed Species on Pt Steps at Low Potentials. *Nat. Commun.* **2022**, *13* (1), 2550.

(4) Xu, Y.; Zhang, L.; Chen, W.; Cui, H.; Cai, J.; Chen, Y.; Feliu, J. M.; Herrero, E. Boosting Oxygen Reduction at Pt(111)|Proton Exchange Ionomer Interfaces through Tuning the Microenvironment Water Activity. *ACS Appl. Mater. Interfaces* **2024**, *16* (4), 4540-4549.

(5) Wang, J. X.; Markovic, N. M.; Adzic, R. R. Kinetic Analysis of Oxygen Reduction on Pt(111) in Acid Solutions: Intrinsic Kinetic Parameters and Anion Adsorption Effects. *J. Phys. Chem. B* **2004**, *108* (13), 4127-4133.

(6) Ganassin, A.; Colic, V.; Tymoczko, J.; Bandarenka, A. S.; Schuhmann, W. Non-Covalent Interactions in Water Electrolysis: Influence on the Activity of Pt(111) and Iridium Oxide Catalysts in Acidic Media. *Phys. Chem. Chem. Phys.* **2015**, *17* (13), 8349-8355.

(7) Shih, A. J.; Arulmozhi, N.; Koper, M. T. M. Electrocatalysis under Cover: Enhanced Hydrogen Evolution Via Defective Graphene-Covered Pt(111). *ACS Catal.* **2021**, *11* (17), 10892-10901.

(8) Nørskov, J. K.; Rossmeisl, J.; Logadottir, A.; Lindqvist, L. Origin of the Overpotential for Orr at a Fuel-Cell Cathode. *J. Phys. Chem. B* **2004**, *108*, 17886-17992.

(9) Peter, A.; de, P. J.; James, K. *Atkins' Physical Chemistry*; Oxford University Press, 2018.

(10) Chen, J.; Fang, L.; Luo, S.; Liu, Y.; Chen, S. Electrocatalytic O2 Reduction on Pt: Multiple Roles of Oxygenated Adsorbates, Nature of Active Sites, and Origin of Overpotential. *J. Phys. Chem. C* **2017**, *121* (11), 6209-6217.

(11) Haynes, W. M. *Crc Handbook of Chemistry and Physics*; CRC press, 2016.

(12) Jinnouchi, R.; Kodama, K.; Hatanaka, T.; Morimoto, Y. First Principles Based Mean Field Model for Oxygen Reduction Reaction. *Phys. Chem. Chem. Phys.* **2011**, *13* (47), 21070-21083.

(13) Wang, V.; Xu, N.; Liu, J. C.; Tang, G.; Geng, W. T. Vaspkit: A User-Friendly Interface Facilitating High-Throughput Computing and Analysis Using Vasp Code. *Comput. Phys. Commun.* **2021**, *267*, 108033.

(14) Rossmeisl, J.; Qu, Z. W.; Zhu, H.; Kroes, G. J.; Nørskov, J. K. Electrolysis of Water on Oxide Surfaces. *J. Electroanal. Chem.* **2007**, *607* (1), 83-89.

(15) Panchenko, A.; Koper, M. T. M.; Shubina, T. E.; Mitchell, S. J.; Roduner, E. Ab Initio Calculations of Intermediates of Oxygen Reduction on Low-Index Platinum Surfaces. *J. Electrochem. Soc.* **2004**, *151* (12), A2016.

(16) Karlberg, G. S.; Rossmeisl, J.; Nørskov, J. K. Estimations of Electric Field Effects on the Oxygen Reduction Reaction Based on the Density Functional Theory. *Phys. Chem. Chem. Phys.* **2007**, *9* (37), 5158-5161.

(17) Tripković, V.; Skúlason, E.; Siahrostami, S.; Nørskov, J. K.; Rossmeisl, J. The Oxygen Reduction Reaction Mechanism on Pt(111) from Density Functional Theory Calculations. *Electrochim. Acta* **2010**, *55* (27), 7975-7981.

(18) Hansen, H. A.; Viswanathan, V.; Nørskov, J. K. Unifying Kinetic and Thermodynamic Analysis of 2 E– and 4 E– Reduction of Oxygen on Metal Surfaces. *J. Phys. Chem. C* **2014**, *118* (13), 6706-6718.

(19) Eslamibidgoli, M. J.; Eikerling, M. H. Electrochemical Formation of Reactive Oxygen Species at Pt (111)—a Density Functional Theory Study. *ACS Catal.* **2015**, *5* (10), 6090-6098.

(20) Liu, S.; White, M. G.; Liu, P. Mechanism of Oxygen Reduction Reaction on Pt(111) in Alkaline Solution: Importance of Chemisorbed Water on Surface. *J. Phys. Chem. C* **2016**, *120* (28), 15288-15298.

(21) Haile, A. S.; Yohannes, W.; Mekonnen, Y. S. Oxygen Reduction Reaction on Pt-Skin Pt3v(111) Fuel Cell Cathode: A Density Functional Theory Study. *RSC Adv.* **2020**, *10* (46), 27346-27356.

(22) Momma, K.; Izumi, F. Vesta 3 for Three-Dimensional Visualization of Crystal, Volumetric and Morphology Data. *J. Appl. Crystallogr.* **2011**, *44* (6), 1272-1276.

(23) Huang, J.; Malek, A.; Zhang, J.; Eikerling, M. H. Non-Monotonic Surface Charging Behavior of Platinum: A Paradigm Change. *J. Phys. Chem. C* **2016**, *120* (25), 13587-13595.

(24) Schmickler, W.; Santos, E. *Interfacial Electrochemistry*; Springer-Verlag Berlin Heidelberg, 2010.

(25) Bockris, J. O. m.; Devanathan, M. A. V.; Müller, K. On the Structure of Charged Interfaces. *Proc. R. Soc. A* **1963**, *274*, 55–79.

(26) Israelachvili, J. N. *Intermolecular and Surface Forces*; Academic Press, 2011.

(27) Martínez-Hincapié, R.; Climent, V.; Feliu, J. M. Peroxodisulfate Reduction as a Probe to Interfacial Charge. *Electrochem. Commun.* **2018**, *88*, 43-46.

(28) Herrero, E.; Mostany, J.; Feliu, J. M.; Lipkowski, J. Thermodynamic Studies of Anion Adsorption at the Pt(111) Electrode Surface in Sulfuric Acid Solutions. *J. Electroanal. Chem.* **2002**, *534* (1), 79-89.

(29) Bard, A. J.; Faulkner, L. R. *Electrochemical Methods Fundamentals and Applications*; John Wiley & Sons, Lnc, 2001.

(30) Zha, Q.-X. *Introduction to Electrode Process Dynamics*; Science Press, 2002.

(31) Zhu, X.; Huang, J.; Eikerling, M. Ph Effects in a Model Electrocatalytic Reaction Disentangled. *JACS Au* **2023**, *3* (4), 1052-1064.
